# Assessing age-friendly urban environments in Indonesia: Validation of the Indonesian age-friendly cities and communities questionnaire (AFCCQ) and differences across urban city categories

**DOI:** 10.1371/journal.pone.0358251

**Published:** 2026-10-08

**Authors:** Nina Kemala Sari, Stepvia Stepvia, Muhana Fawwazy Ilyas

**Affiliations:** 1 Geriatric Division, Department of Internal Medicine, Dr. Cipto Mangunkusumo National Referral Hospital, Faculty of Medicine, Universitas Indonesia, Jakarta, Indonesia; 2 Faculty of Medicine, Universitas Sebelas Maret, Surakarta, Indonesia; Khoy University of Medical Sciences, IRAN, ISLAMIC REPUBLIC OF

## Abstract

**Background/Objectives:**

The global aging population is growing rapidly, especially in Southeast Asia and Indonesia. This demographic shift poses significant challenges for urban planning and public health, as older adults face barriers to accessing essential services and engaging in their communities. This study assesses the age-friendliness of urban environments in Indonesia by evaluating the psychometric properties of an Indonesian-language version of the Age-Friendly Cities and Communities Questionnaire (AFCCQ) and comparing perceived age-friendliness across urban categories.

**Methods:**

A cross-sectional survey using the Indonesian version of the AFCCQ was conducted between 7 January and 3 February 2025. The AFCCQ covers nine domains: housing, social participation, respect and social inclusion, civic participation and employment, communication and information, community support and health services, outdoor spaces and buildings, transportation, and financial status. Validity was assessed using item–total Spearman correlations and exploratory factor analysis; reliability was assessed using Cronbach’s α. Differences in domain scores across five urban categories were examined using the Kruskal–Wallis test with Bonferroni adjustment across the nine domain-level tests (α = 0.05/9 = 0.0056).

**Results:**

The study involved 505 community-dwelling older adults aged ≥60 years, with a mean age of 69.4 years; 81.6% were female. The average AFCCQ score was 14.96 ± 10.46. Overall Cronbach’s α was 0.90. The KMO value was 0.83 and Bartlett’s test was significant (χ²(253) = 13,374.41, p < 0.001); the first unrotated component accounted for 35.92% of the variance, although 7 components had eigenvalues greater than 1. Nominally significant differences across city categories were observed in housing, social participation, and transportation (p < 0.05); none remained significant after Bonferroni adjustment for nine domain-level comparisons. These comparisons are reported as exploratory.

**Conclusion:**

The study indicates that the Indonesian AFCCQ has acceptable internal consistency. Item-level convergence was weak for the two respect and social inclusion items, and the exploratory factor analysis did not reproduce the nine-domain structure. Exploratory differences across urban categories suggest that perceived age-friendliness may vary by city size, particularly for housing, social participation, and transportation, and provide a basis for further investigation in representative samples. Confirmatory factor analysis and formal test–retest assessment in an independent Indonesian sample remain necessary.

## Introduction

The global population of older adults is growing rapidly. The World Health Organization (WHO) estimates that the number of people aged 60 years and over will rise from 1 billion in 2020 to 1.4 billion by 2030, by which time one in six people worldwide will be aged 60 or over; between 2015 and 2050 the proportion of the world’s population aged over 60 will nearly double, from 12% to 22% [1]. This demographic transition is anticipated to be predominantly focused in urban areas [2]. Southeast Asia is witnessing significant growth in its aging demographic, with the proportion of individuals aged 60 or older projected to rise from 9.8% in 2017 to 13.7% in 2030 and 20.3% by 2050 [3]. Indonesia, like many other nations, is currently undergoing a demographic shift. Due to improvements in life expectancy, Indonesia’s older adults are anticipated to grow by 20.5 million (a 173% increase) from 2010 to 2035 [4]. The proportion of Indonesians residing in urban areas is projected to rise from 50% in 2010 to 67% in 2035, attributable to population growth, rural-to-urban migration, and changes in urban classification [5]. With rising life expectancy, older adults face numerous health and quality-of-life concerns, including non-communicable diseases, mental health disorders, dementia, injuries, and physical limitations [6].

In 2007, the World Health Organization (WHO) launched the Age-Friendly Cities and Communities (AFCC) global initiative to address the needs of older persons and create urban environments conducive to aging populations [7]. In 2010, the WHO launched the Global Network for AFCC to promote the exchange of information and learning among cities and communities globally [8]. This network provides a platform for cities and communities to share ideas and experiences related to age-friendly initiatives [9]. Furthermore, the WHO has published guidelines for developing age-friendly cities, including a checklist of essential characteristics. These guidelines were formulated based on contributions from 33 cities across 22 countries included in the WHO Global Age-Friendly Cities project consultation. The WHO AFC framework includes eight key domains: outdoor spaces and buildings; transportation; housing; social participation; respect and social inclusion; civic participation and employment; communication and information; and community support and health services [10]. These guidelines serve as a tool for assessing and enhancing the age-friendliness of urban environments and for mitigating social exclusion among older adults [8].

In assessing urban buildings and outdoor environments, prior studies have shown that older adults are concerned about the lack of auditory guidance at pedestrian crossings, the absence of separated bicycle lanes adjacent to sidewalks, and the lack of dedicated queues for older adults in public spaces [11]. Traditionally, older adults in Asia were cared for by younger family members, particularly when seriously ill or unable to perform self-care. Declining fertility rates, rural-to-urban migration, increased female workforce participation, and evolving views of filial piety have reduced multi-generational cohabitation [12].

The global adoption of the WHO guidelines illustrates their potential as a tool for promoting inclusion of older adults, a fundamental aspect of creating supportive environments for aging populations [13]. Research has linked age-friendly environments to active lifestyles among older adults. A study in Israel emphasized that accessible public transportation encourages active engagement among older adults [14]. Canadian research showed that older persons who can drive are over twice as likely to engage in social and out-of-home activities than non-drivers [15]. In Hong Kong, transportation and respect/social inclusion were among the strongest determinants of self-rated health and community belonging [16]. Behavioural factors such as a nutritious diet and physical activity are associated with active aging [17], and mental stimulation through intellectual activities is linked to better cognitive function in older adults [18].

The Age-Friendly Cities and Communities Questionnaire (AFCCQ) is a quantitative tool for assessing age-friendliness. It was developed by Dikken et al. in The Hague in 2020 and consists of 23 items across nine domains based on the WHO framework, with the financial situation domain added to the eight WHO domains [19]. Unlike the WHO Checklist, the AFCCQ is a structured short-form instrument that supports cross-cultural comparison [20]. The original AFCCQ is available in Dutch and British English, and validated versions exist in several languages including Turkish [21], Romanian [22] and Polish [23], among others.

To build on this framework, this study evaluates older adults’ experiences with age-friendly urban environments in Indonesia using the Indonesian version of the AFCCQ. We report the translation and cultural-adaptation procedure, psychometric evidence (content validity, item–total correlations, exploratory factor analysis, internal consistency), and comparisons of perceived age-friendliness across five Indonesian urban categories.

## Materials and methods

### Research design and ethics

This cross-sectional study used both online and offline methodologies to assess the age-friendliness of multiple urban areas in Indonesia, utilising the AFCCQ. The questionnaire comprises 23 items across nine domains established by the WHO. Data collection was carried out from 7 January 2025–3 February 2025. The study adhered to the STROBE reporting checklist for observational research.

Ethical approval for the study was granted by the Ethics Committee of the Faculty of Medicine, Universitas Indonesia – Dr. Cipto Mangunkusumo National Referral Hospital on 6 January 2025 (approval letter no. KET-19/UN2.F1/ETIK/PPM.00.02/2025; protocol no. 24-10-1617). The study was conducted in accordance with the Declaration of Helsinki. Written informed consent was obtained from every participant prior to data collection. For online recruitment, consent was recorded electronically as the first item of the Google Form. For offline recruitment, consent was documented on a paper form signed in the presence of a trained research assistant. For participants who were unable to read or write, the study procedures and consent form were read aloud in plain language by a trained assistant; consent was then provided in writing by a legally authorized representative (spouse, adult child, or legal guardian) after the participant confirmed verbal assent. All responses were de-identified at the point of data entry. The de-identified dataset supporting this article has been deposited in a public repository (see Data Availability).

### Setting and recruitment

Offline data collection was conducted at two sites in Jakarta: Dr. Cipto Mangunkusumo National Referral Hospital (RSCM), Indonesia’s national tertiary referral and teaching hospital, and the Paris Community Health Center, a primary-care facility. The two sites were chosen to combine national-referral and community-level recruitment within the metropolitan and megapolitan urban categories. Online recruitment was carried out through the 2025 National Senior Expo: a dedicated booth and a QR code directed interested older adults to a Google Form containing the questionnaire and electronic informed consent. For participants with limited literacy or visual impairment, trained research assistants read items verbatim and recorded responses without interpretation.

### Participants and sampling

Participants were older adults aged 60 years and above residing in one of five city categories defined by Indonesian Government Regulation No. 26 of 2008: small, medium-sized, large, metropolitan, and megapolitan. A purposive sampling approach was used. Individuals with confirmed cognitive impairment, based on medical history or clinical evaluation, were excluded. All participants provided written informed consent before participation. Exclusion for cognitive impairment was based on documented medical history and/or clinical evaluation by the recruiting clinician only. No minimum duration of residence in the city was required for eligibility, and no residence-duration criterion was applied.

### Urban setting and city characteristics

Indonesia’s spatial planning regulation distinguishes five types of urban areas (small, medium-sized, large, metropolitan, and megapolitan) based on population size and functional role within the national settlement hierarchy [24]. In this study, older adults were recruited from 93 distinct locations, comprising municipalities (kota), regencies (kabupaten), district towns and sub-district settlements, across multiple provinces, which were classified according to Indonesian Government Regulation No. 26/2008 and recent population estimates.

The megapolitan category was dominated by the Greater Jakarta (Jabodetabek) conurbation, including the Special Capital Region of Jakarta and surrounding cities (Depok, Bekasi, Bogor, Tangerang, South Tangerang). Jakarta megapolitan alone contributes around 20.85% of Indonesia’s Gross Regional Domestic Product [25]. Metropolitan cities included Bandung, Medan, Denpasar, Pontianak, Pekanbaru, Palangka Raya, and Surakarta (city and surrounding population > 1 million). Large cities included Cilegon, Tangerang City, Bogor City, and other urban centres in West and Central Java (population > 500,000). Medium-sized cities (population 100,000–500,000) included Purworejo, Kulon Progo, Rembang, Banyumas, Tuban, and other district capitals. Small cities (population < 100,000) were represented by smaller district towns and peri-urban settlements such as parts of Trucuk, Sarang, Tegowanu, Sumpiuh, and several village-based townships [24, 26]. A complete list is provided in Supporting Information (S1 Table).

### Translation and cultural adaptation

The original AFCCQ, developed by Dikken et al. (2020), comprises 23 items rated on a 5-point Likert scale from −2 (strongly disagree) to +2 (strongly agree), with higher scores indicating greater perceived age-friendliness [19]. The questionnaire covers nine domains: outdoor spaces and buildings; transportation; housing; social participation; respect and social inclusion; civic participation and employment; communication and information; community support and health services; and financial situation. The AFCCQ is distributed under a Creative Commons Attribution licence via the WHO Age-Friendly World platform; copyright remains with the developers [19].

Following WHO/ISPOR good-practice guidelines and the AFCCQ developers’ guidance, we implemented a standardized five-step process: (1) dual independent forward translations (English→Indonesian); (2) reconciliation; (3) back-translation by a blinded translator; (4) expert committee review (geriatric medicine, urban/public health, psychometrics, survey methodology); and (5) cognitive debriefing with older adults from the target population to confirm clarity, relevance, and cultural appropriateness. Minor wording refinements were made before fielding the final Indonesian AFCCQ.

Content validity was assessed by calculating the proportion of experts who rated each item as relevant (scores 3–4 on a 4-point relevance scale). All 23 items were rated as relevant by at least 4 of 5 experts (≥ 80% agreement). Nineteen items achieved complete agreement (5/5), and four items achieved 4/5 agreement; minor wording refinements were made for these four items based on qualitative comments. Item-level content validity indices (I-CVI) ranged from 0.80 to 1.00; the scale-level average (S-CVI/Ave) was 0.97.

The full Indonesian-language AFCCQ is provided as Supporting Information (S2 File) under a Creative Commons Attribution (CC BY) license. The original English version is publicly available via the WHO Age-Friendly World platform.

### Questionnaire validation

According to Donner and Eliasziw [27] and Mundfrom et al. [28], a sample size of at least 100 participants is considered sufficient for reliability studies and factor analysis. In this study, the main validation sample comprised 505 older adults from urban areas in Indonesia who provided informed consent and met the age ≥ 60 eligibility criterion. Individuals with cognitive impairment, confirmed by medical history or clinical evaluation, were excluded. Participants completed the questionnaire on-site or online under supervision.

### Statistical analysis

Statistical analyses were performed using IBM SPSS Statistics version 27.0 (IBM Corp., Armonk, NY, USA). All analyses reported were additionally re-executed in Python 3 (pandas, NumPy, SciPy). Descriptive statistics included means and standard deviations for continuous data and frequencies and percentages for categorical variables; because the group comparisons are rank-based, medians and interquartile ranges (IQR) are also reported for domain and total scores. The questionnaire results were categorized by functional domain and evaluated for validity and reliability. Item–total correlations were computed as corrected item–total Spearman correlations: each item was correlated with the sum of the remaining 22 items, excluding the indexed item, so that the item under evaluation does not contribute to the total against which it is assessed [29]. Exploratory factor analysis was conducted to evaluate construct validity [30]. The Kaiser–Meyer–Olkin value (> 0.5) was calculated to evaluate sampling adequacy, and data factorability was assessed using Bartlett’s test of sphericity. The number of extracted factors was determined by the scree plot and by eigenvalues greater than 1 [31].

Reliability was evaluated using Cronbach’s α, with values of 0.7 or above signifying satisfactory internal consistency (α < 0.5 unacceptable; 0.5 ≤ α < 0.6 poor; 0.6 ≤ α < 0.7 questionable; 0.7 ≤ α < 0.8 acceptable; 0.8 ≤ α < 0.9 good; α ≥ 0.9 excellent) [32]. Differences in AFCCQ scores across the five city categories were assessed using the Kruskal–Wallis H test for data that did not meet normality assumptions; a Bonferroni-adjusted significance threshold was applied across the family of nine domain-level tests (0.05/9 = 0.0056). Dunn’s post-hoc pairwise comparisons were prespecified to follow a significant omnibus test; because no domain-level test met the family-wise threshold, pairwise comparisons were not performed. A two-sided p-value below 0.05 was considered nominally significant, and only results below 0.0056 are interpreted as statistically significant after adjustment for multiplicity. Horn’s parallel analysis (1,000 permutations, 95th-percentile criterion) was performed and is reported alongside the eigenvalue and scree-plot criteria. All psychometric procedures were aligned with COSMIN recommendations, and administration/scoring adhered to the AFCCQ developers’ instructions.

## Results

### Socio-demographic profile

A total of 505 individuals participated in the study. Table 1 delineates the demographic and socioeconomic attributes of the study participants. The majority were female (412, 81.6%), with a mean age of 69.4 ± 6.3 years (median 68, IQR 64–73, range 60–90). The majority of respondents held an associate degree (151, 29.9%), with smaller proportions holding a bachelor’s degree (102, 20.2%) or higher qualifications. Most respondents (367, 72.7%) lived with their spouses, 88 (17.4%) with their children, and 23 (4.6%) alone. Regarding urban setting, 219 participants (43.4%) resided in megapolitan areas and 166 (32.9%) in metropolitan cities, predominantly in Greater Jakarta. The remaining 56 (11.1%), 52 (10.3%), and 12 (2.4%) respondents lived in large, medium-sized, and small cities, respectively.

**Table 1:** Socioeconomic and demographic characteristics of study participants.

Characteristics	Classification	n (%) (N = 505)
Sex	Male	93 (18.4)
	Female	412 (81.6)
Age (years), mean (SD)		69.4 (6.3)
Age (years), median (IQR)		68 (64–73)
Education	No formal education	9 (1.8)
	Elementary school graduate	66 (13.1)
	Junior high school graduate	21 (4.2)
	Senior high school graduate	122 (24.2)
	Associate graduate	151 (29.9)
	Bachelor’s degree	102 (20.2)
	Master’s degree	29 (5.7)
	Doctoral degree	5 (1.0)
Place of residence	Small city	12 (2.4)
	Medium-sized city	52 (10.3)
	Large city	56 (11.1)
	Metropolitan city	166 (32.9)
	Megapolitan city	219 (43.4)
Reside with	Spouse	367 (72.7)
	Sibling(s)	7 (1.4)
	Relative(s)	2 (0.4)
	Child(ren)	88 (17.4)
	Grandchild(ren)	2 (0.4)
	Caregiver	3 (0.6)
	Living alone	23 (4.6)
	Other	13 (2.6)

### AFCCQ responses

The overall mean AFCCQ score was 14.96 ± 10.46 (median 16, IQR 10–21), suggesting that respondents had a moderate perception of age-friendliness. Mean AFCCQ scores across functional domains are stated in Table 2. In the housing domain, the mean score was 2.07 ± 1.41. In the social participation domain, the mean score was 2.90 ± 3.26. In the respect and social inclusion domain, the mean score was −0.93 ± 1.81. In the civic participation and employment domain, the mean score was 1.55 ± 1.41. In the communication and information domain, the mean score was 1.65 ± 1.28. In the community support and health services domain, the mean score was 4.35 ± 2.96. In the outdoor spaces and buildings domain, the mean score was 1.37 ± 1.70. In the transportation domain, the mean score was 0.97 ± 1.90. In the financial situation domain, the mean score was 1.02 ± 1.86. The respect and social inclusion domain was the only domain with a negative mean score, indicating that this was the least favourably rated aspect of age-friendliness in this sample.

**Table 2:** AFCCQ item, domain and total scores.

Domain	Item	Mean ± SD	Median (IQR)
Housing	Q1	1.04 ± 0.74	1 (1 –1)
	Q2	1.04 ± 0.72	1 (1 –1)
	Total	2.07 ± 1.41	2 (2 –2)
Social participation	Q3	0.72 ± 0.87	1 (0–1)
	Q4	0.74 ± 0.85	1 (0–1)
	Q5	0.72 ± 0.83	1 (0–1)
	Q6	0.72 ± 0.84	1 (0–1)
	Total	2.90 ± 3.26	4 (0–4)
Respect and social inclusion	Q7	−0.38 ± 0.97	−1 (−1–0)
	Q8	−0.56 ± 0.95	−1 (−1–0)
	Total	−0.93 ± 1.81	−2 (−2–0)
Civic participation and employment	Q9	0.78 ± 0.76	1 (0–1)
	Q10	0.77 ± 0.76	1 (0–1)
	Total	1.55 ± 1.41	2 (1–2)
Communication and information	Q11	0.82 ± 0.68	1 (1 –1)
	Q12	0.83 ± 0.67	1 (1 –1)
	Total	1.65 ± 1.28	2 (1 –2)
Community support and health services	Q13	0.85 ± 0.69	1 (1 –1)
	Q14	0.91 ± 0.65	1 (1 –1)
	Q15	0.88 ± 0.72	1 (1 –1)
	Q16	0.87 ± 0.71	1 (1 –1)
	Q17	0.84 ± 0.71	1 (1 –1)
	Total	4.35 ± 2.96	5 (3 –5)
Outdoor spaces and buildings	Q18	0.71 ± 0.86	1 (0–1)
	Q19	0.67 ± 0.86	1 (0–1)
	Total	1.37 ± 1.70	2 (0–2)
Transportation	Q20	0.49 ± 0.96	1 (0–1)
	Q21	0.48 ± 0.95	1 (0–1)
	Total	0.97 ± 1.90	2 (0–2)
Financial situation	Q22	0.51 ± 0.93	1 (0–1)
	Q23	0.51 ± 0.93	1 (0–1)
	Total	1.02 ± 1.86	2 (0–2)
Overall total		14.96 ± 10.46	16 (10 –21)

### Validity of the questionnaire

A satisfactory level of agreement among respondents was used to establish the questionnaire’s content and face validity. Based on the item–total Spearman correlation analysis (Table 3), 21 of the 23 items correlated significantly and positively with the sum of the remaining items (ρ = 0.03–0.60). The two respect and social inclusion items were the exception: Q7 (ρ = 0.08, p = 0.086) and Q8 (ρ = 0.03, p = 0.496) did not correlate significantly with the remainder of the scale. The Bartlett test of sphericity (χ²(253) = 13,374.41, p < 0.01) confirmed the factorability of the correlation matrix, while the Kaiser–Meyer–Olkin (KMO) test value of 0.83 indicated adequate sampling adequacy. The scree plot (Fig 1) showed a pronounced break after the first component, whose eigenvalue was 8.26 and which accounted for 35.92% of the variance. Applying the eigenvalue > 1 criterion stated in the Methods, however, retained 7 components rather than one, and Horn’s parallel analysis retained six. The retention criteria therefore did not converge on a unidimensional solution, and the nine-domain structure of the original instrument was not reproduced in these data. In the one-component solution 17 of the 23 items loaded at or above 0.40; the four social participation items (Q3–Q6, 0.29–0.37) and the two respect and social inclusion items (Q7, 0.09; Q8, 0.05) fell below this threshold.

**Table 3:** Corrected item–total Spearman correlations between AFCCQ items and the sum of the remaining items.

Domain	Item	ρ (corrected)	p-value
Housing	Q1	0.43	<0.001
	Q2	0.39	<0.001
Social participation	Q3	0.35	<0.001
	Q4	0.45	<0.001
	Q5	0.44	<0.001
	Q6	0.44	<0.001
Respect and social inclusion	Q7	0.08	0.086
	Q8	0.03	0.496
Civic participation and employment	Q9	0.54	<0.001
	Q10	0.48	<0.001
Communication and information	Q11	0.53	<0.001
	Q12	0.50	<0.001
Community support and health services	Q13	0.54	<0.001
	Q14	0.60	<0.001
	Q15	0.56	<0.001
	Q16	0.54	<0.001
	Q17	0.56	<0.001
Outdoor spaces and buildings	Q18	0.58	<0.001
	Q19	0.55	<0.001
Transportation	Q20	0.51	<0.001
	Q21	0.51	<0.001
Financial situation	Q22	0.53	<0.001
	Q23	0.53	<0.001

**Fig 1:**
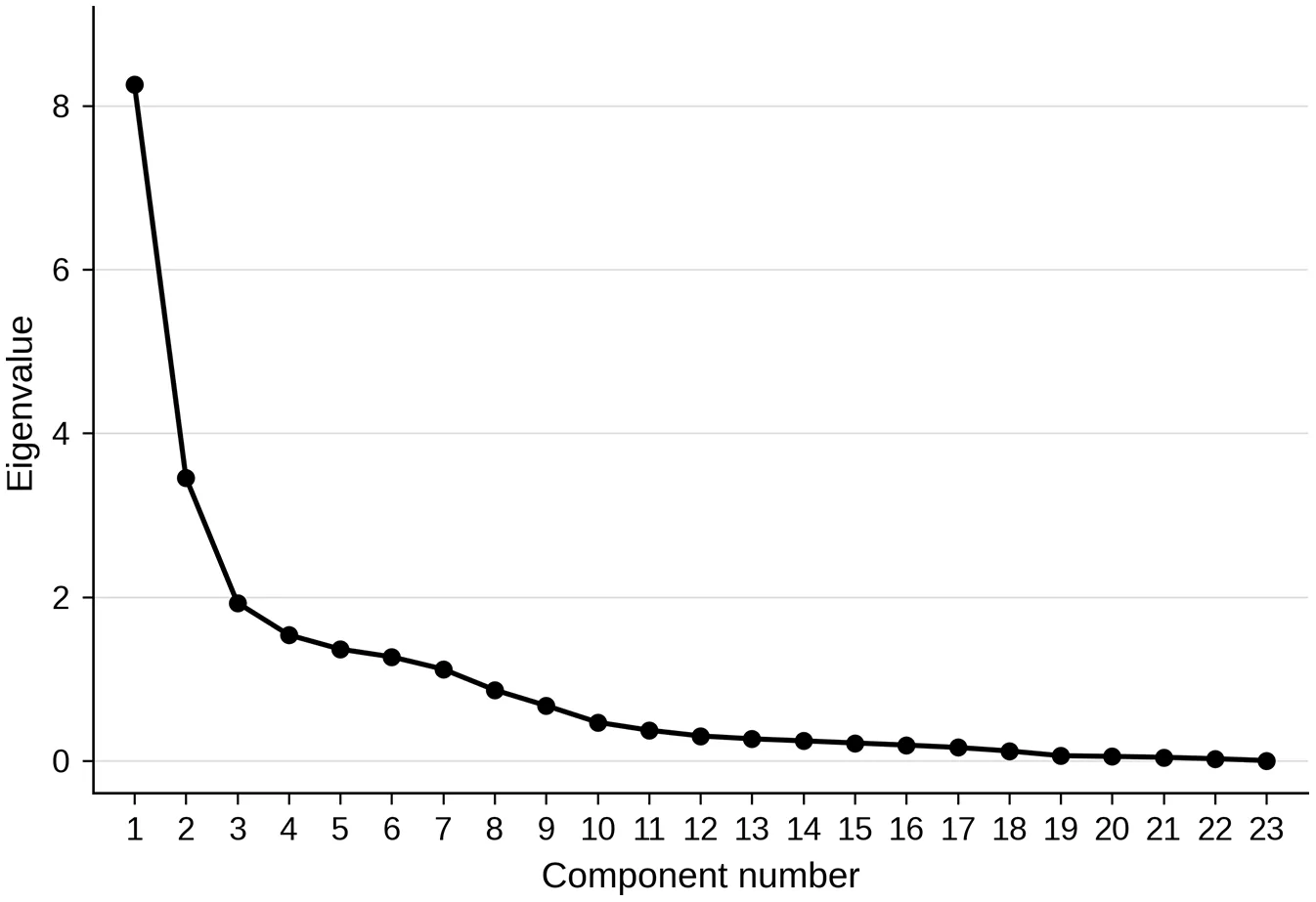
AFCCQ scree plot.

### Reliability of the questionnaire

The questionnaire demonstrated good internal consistency, as shown by a Cronbach’s α coefficient of 0.90. The internal consistency within the individual domains ranged from 0.83 to 1.00 (Table 4), with the highest reliability in the financial situation domain (α = 1.00). Domain-level α values are given in Table 4. The very high coefficients in several two-item domains require cautious interpretation rather than being read as evidence of strong reliability. Inspection of the item-level data shows that the paired items in these domains were answered identically by almost all respondents: Q22 and Q23 agreed exactly for 99.6% of participants (ρ = 1.00); Q20 and Q21 agreed exactly for 96.0% of participants (ρ = 0.96); Q4 and Q5 agreed exactly for 94.7% of participants (ρ = 0.93). Coefficients derived from near-duplicate items reflect item overlap rather than independent measurement of the underlying construct, and the two financial items in particular behave as a single repeated question.

**Table 4:** Internal consistency of the AFCCQ.

Domain (items)	No. of items	Cronbach’s α	ICC*
Overall (Q1–Q23)	23	0.90	0.87
Housing (Q1–Q2)	2	0.93	
Social participation (Q3–Q6)	4	0.97	
Respect and social inclusion (Q7–Q8)	2	0.88	
Civic participation and employment (Q9–Q10)	2	0.83	
Communication and information (Q11–Q12)	2	0.88	
Community support and health services (Q13–Q17)	5	0.91	
Outdoor spaces and buildings (Q18–Q19)	2	0.97	
Transportation (Q20–Q21)	2	0.98	
Financial situation (Q22–Q23)	2	1.00	

### Differences in AFCCQ scores across city categories

Nominally significant differences in AFCCQ scores were found across city categories for the housing (p = 0.038), social participation (p = 0.036), transportation (p = 0.033) (Table 5). None of the nine domain-level comparisons met the Bonferroni-adjusted threshold of 0.05/9 = 0.0056, and the omnibus comparison of total AFCCQ scores was not significant even at the nominal level (H = 7.53, p = 0.110). Because no domain survived adjustment for multiplicity, prespecified Dunn pairwise comparisons were not performed, and these differences are reported as exploratory and hypothesis-generating rather than as confirmed effects of city category. Differences in other domains, such as respect and social inclusion, civic participation and employment, communication and information, community support and health services, outdoor spaces and buildings, and financial situation, were not statistically significant at either threshold.

**Table 5:** Differences in AFCCQ scores across city categories.

Domain (items)	Small city(n = 12)	Medium-sized city(n = 52)	Large city(n = 56)	Metropolitan city(n = 166)	Megapolitan city(n = 219)	H	p-value
Overall (Q1–Q23)	20 (15 –24)	16 (10 –22)	19 (11 –23)	15 (8 –20)	16 (10 –20)	7.53	0.110
Housing (Q1–Q2)	2 (2 –2)	2 (2 –2)	2 (2 –4)	2 (2 –2)	2 (2 –2)	10.15	0.038*
Social participation (Q3–Q6)	4 (3 –4)	3 (0–4)	4 (0–4)	4 (2 –4)	4 (2 –4)	10.31	0.036*
Respect and social inclusion (Q7–Q8)	−0 (−2–2)	−2 (−2–0)	0 (−2–2)	−2 (−2–0)	−2 (−2–0)	4.54	0.338
Civic participation and employment (Q9–Q10)	2 (2 –2)	2 (1 –2)	2 (1 –2)	2 (1 –2)	2 (1 –2)	8.34	0.080
Communication and information (Q11–Q12)	2 (2 –2)	2 (1 –2)	2 (2 –2)	2 (1 –2)	2 (1 –2)	7.39	0.117
Community support and health services (Q13–Q17)	5 (5 –5)	5 (4 –5)	5 (5 –5)	5 (3 –5)	5 (3 –5)	8.18	0.085
Outdoor spaces and buildings (Q18–Q19)	2 (2 –2)	2 (0–2)	2 (2 –2)	2 (0–2)	2 (0–2)	4.87	0.301
Transportation (Q20–Q21)	2 (2 –2)	2 (0–2)	2 (0–2)	2 (0–2)	2 (0–2)	10.51	0.033*
Financial situation (Q22–Q23)	2 (2 –2)	2 (0–2)	2 (0–2)	2 (0–2)	2 (0–2)	7.25	0.123

Kruskal–Wallis test, df = 4. Data are presented as median (interquartile range), because the test is rank-based. *nominally significant at p < 0.05. No domain met the Bonferroni-adjusted threshold of 0.05/9 = 0.0056.

## Discussion

This study involved adapting and validating the AFCCQ for older adults in Indonesia. This instrument, initially created by Dikken et al. in 2020, is based on the WHO age-friendly cities framework and is disseminated through the WHO Age-Friendly World platform as a tool for evaluating the perceived age-friendliness of urban communities [19]. The AFCCQ follows a citizen-centered approach, aiming to capture the perspectives of older adults and communicate them to policymakers and local authorities. Researchers and practitioners can use the AFCCQ to evaluate the age-friendliness of a city or community, track changes over time, and assess the effectiveness of policies and social programs [22].

The validation segment of the AFCCQ included translation, face validity assessment, and content validity evaluation within the context of Indonesia’s older population. This study utilized a sampling method that encompassed individuals from many cities in Indonesia, representing a range of socio-demographic profiles. The sample consisted of respondents from small cities (2.4%), medium-sized cities (10.3%), large cities (11.1%), metropolitan cities (32.9%), and megapolitan cities (43.4%). This distribution reflects the urban-dominant residential pattern of older adults in Indonesia; however, smaller cities remain underrepresented and the sample is not population-representative.

Evaluating age-friendly urban policy requires specific methodologies to assess the age-friendliness of different urban settings. Before using the AFCCQ to gather data, researchers in other countries need to evaluate its cultural adaptation, especially when working with cultures that differ significantly from Dutch or Western European norms. Several studies from Western countries have highlighted the importance of adapting the AFCCQ to align with local priorities. A study conducted in Australia highlighted essential parameters for age-friendly development at the local government level in South Australia. The research indicated that the WHO age-friendly indicators are comprehensive and require customization to meet local needs [33].

Comparison with the original development study and with other AFCCQ validations helps to situate these findings. Dikken et al. developed the AFCCQ in The Hague using a COSMIN-based procedure, reducing an initial pool of 64 candidate questions to the final 23 items and establishing the nine-domain structure that the instrument is intended to measure [19]. Our sample differs from that development sample and from most subsequent validations in three respects that plausibly bear on the results: it was recruited purposively rather than as a representative city sample; it was strongly female-predominant (81.6% women); and it was drawn from many locations spread across five urban categories rather than from a single city. The Turkish validation by Özer et al. reported acceptable internal consistency for the Turkish version [21], and the Romanian and Polish applications by Ivan et al. and Perek-Białas et al. both used the instrument to characterise subgroups of older citizens within individual cities [22, 23], and the Hebrew validation in Israel reported acceptable psychometric performance in a further cultural setting [34]. The AFCCQ has also been applied outside Europe, to the age-friendliness of Japanese municipalities [35].

Two differences from these studies stand out in our data. First, our respect and social inclusion domain was the only domain with a negative mean score (−0.93), and its two items did not converge with the rest of the scale; this domain has not been reported as the weakest in the Dutch, Turkish, Romanian or Polish work, and the discrepancy may reflect culturally specific interpretation of respect and inclusion items in the Indonesian context, or wording introduced during translation. Second, our exploratory analysis did not recover the nine-domain structure, whereas the original development work established it on an independent and more representative sample using a different analytical route. Differences in recruitment, in the age and sex composition of the samples, and in the extraction and retention procedures applied are all plausible contributors, and we cannot distinguish between them with the present design. The AFCCQ developers make a validation manual available on request and recommend that new language versions follow the COSMIN protocol; future Indonesian work should be conducted explicitly against that guidance, with the development team consulted at the translation stage.

A satisfactory level of consensus among panelists confirmed the questionnaire’s content and face validity in the current study. The item–total analysis indicated that 21 of 23 items converged acceptably with the remainder of the scale, while the two respect and social inclusion items (Q7, Q8) did not correlate significantly with it and should be revised or replaced. The Bartlett test of sphericity (χ²(253) = 13,374.41, p < 0.01) validated the factorability of the correlation matrix, and the Kaiser–Meyer–Olkin (KMO) value of 0.83 indicated sufficient sample adequacy. The first component was dominant (eigenvalue 8.26, 35.92% of variance), but 7 components exceeded an eigenvalue of 1 and parallel analysis retained six, so the data neither confirm unidimensionality nor reproduce the nine-domain structure. Cronbach’s α coefficient was 0.90, signifying strong internal consistency at the scale level, although the domain-level coefficients are inflated by the near-duplicate item pairs described above.

This study found nominal differences in AFCCQ scores among cities in the domains of housing (Q1–Q2), social participation (Q3–Q6), and transportation (Q20–Q21), none of which survived adjustment for multiple comparisons. Housing accessibility is critical for older adults as it affects their ability to live independently and safely. The observed pattern does not follow city size monotonically: the highest mean housing score was recorded in large cities (2.50) and the lowest in metropolitan cities (1.92), with small cities (2.33) scoring above both metropolitan and megapolitan areas (2.07). Because this study did not measure housing policy, infrastructure investment or built-environment characteristics, we cannot attribute the differences to those factors; indicators for infrastructure provision in Indonesian urban housing have been described elsewhere [36].

Social participation is crucial for enhancing the quality of life in older persons by fostering a sense of connection and belonging. Social participation includes attending local gatherings, cultural events, and recreational activities; volunteering; and interacting with family and friends. A strong sense of belonging within neighborhoods supports higher levels of social participation. In this sample the highest mean social participation score was observed in metropolitan cities (3.35) and the lowest in large cities (2.18). This ordering does not correspond to a simple urban–rural or size gradient, and the present cross-sectional design cannot establish why it arises [37–39]. The availability of diverse public amenities, combined with high-density neighborhoods and good connectivity, encourages shared use of common facilities and fosters a stronger sense of community [40, 41]. A prior AFCCQ study has highlighted community programs that promote social participation and civic engagement among older adults [23].

Transportation access is a key factor influencing the independence and mobility of older adults. Public transportation enables older adults to access healthcare services, participate in social activities, and maintain their autonomy. In this sample, however, the highest mean transportation score was recorded in small cities (1.50) and the lowest in metropolitan cities (0.68); megapolitan and large cities were intermediate (1.18 and 1.18 respectively). This does not match the expectation that larger cities offer better perceived transport access [42, 43], though the comparison did not survive correction for multiplicity and the small-city group comprised only 12 participants. Even where urban transportation networks are accessible in principle, many transit stations and facilities remain difficult to navigate for older adults with mobility impairments [44]. Inadequate funding in various low- and middle-income countries acts as a barrier to the implementation of inclusive transportation interventions; identifying low-cost sustainable solutions is therefore important [45].

Overall, the Indonesian AFCCQ demonstrated acceptable internal consistency, consistent with the WHO age-friendly cities framework. The domains rated least favourably — respect and social inclusion, transportation, and financial situation — indicate where perceived age-friendliness was weakest in this sample. These exploratory differences require confirmation in larger and more representative samples before they can guide policy.

### Limitations

This study has several limitations. First, the use of purposive sampling and a female-predominant sample may limit generalizability. Second, the cross-sectional design precludes causal or temporal inference. Third, reliance on self-reported data may introduce recall and social desirability biases. Fourth, the very high internal consistency observed in several two-item domains reflects near-duplicate responding rather than independent measurement: the two financial items were answered identically by 99.6% of participants. These item pairs require revision. Fifth, although this study covered five urban categories, smaller cities remain underrepresented (small-city n = 12), so between-category contrasts should be regarded as exploratory; future research should include a broader range of geographic settings to enhance external validity. Sixth, the exploratory factor analysis did not reproduce the nine-domain structure, and the eigenvalue, scree and parallel-analysis criteria did not converge; confirmatory factor analysis in an independent sample is needed to test the latent structure formally. Seventh, although nominal differences were observed across city categories, none of the p-values survived Bonferroni adjustment across the nine domain-level tests.

## Conclusion

This study provides evidence that the AFCCQ is a psychometrically promising instrument for evaluating perceived age-friendliness of urban environments for older adults in Indonesia. Internal consistency at the scale level was good, but item-level convergence was weak for the respect and social inclusion domain, several two-item domains contained near-duplicate items, and the exploratory factor analysis did not reproduce the instrument’s nine-domain structure. Differences across city categories were nominal only and did not survive adjustment for multiple comparisons, so this study does not establish that perceived age-friendliness differs by city category; the observed patterns are hypothesis-generating. Confirmatory psychometric work, refinement of the weakly-converging respect items and the near-duplicate item pairs, documented test–retest assessment, and replication in more representative samples are recommended before the Indonesian AFCCQ is used to evaluate the impact of urban policy on perceived age-friendliness.

## Supporting information

S1 Table Distribution of participants across urban categories, cities and districts. Number and percentage of participants recruited in each city or district, grouped by the five urban categories defined in Indonesian Government Regulation No. 26 of 2008.
(DOCX)

S1 File Age-Friendly Cities and Communities Questionnaire (AFCCQ) – Indonesian version. Indonesian-language AFCCQ administered in this study, together with the original English version and the developers’ scoring instructions, reproduced under a Creative Commons Attribution (CC BY) licence.
(PDF)
