# Supplementary material for: Assessing age-friendly urban environments in Indonesia: Validation of the Indonesian age-friendly cities and communities questionnaire (AFCCQ) and differences across urban city categories
Source: PLoS One. 2026 Oct 8;21(10):e0358251. doi: 10.1371/journal.pone.0358251 (PMC13648996; doi:10.1371/journal.pone.0358251)
Supplement: Supplementary file 1 [file pone.0358251.s002.pdf]

The Age-Friendly Cities and Communities Questionnaire (AFCCQ) used in this study is adapted from the original English version developed by J. Dikken, R.F.M. van den Hoven, W.H. van Staalduinen, L.M.T. Hulsebosch-Janssen, and J. van Hoof, published in the International Journal of Environmental Research and Public Health (2020; 17(18):6867. https://doi.org/10.3390/ijerph17186867), and made available through the World Health Organization's Age-Friendly World platform (https://extranet.who.int/agefriendlyworld/afp/the-age-friendly-cities-and-communities-questionnaire-afccq/).

The Indonesian version was translated by a certified sworn translator and is provided here under the Creative Commons Attribution (CC BY) license. All original sources are fully acknowledged, and this translated version is shared with permission for use in open-access research and policy development.

Kuesioner Kota dan Komunitas Ramah Usia AFCCQ [Bahasa Inggris]

Item	Domain	Sangat tidak setuju	Tidak setuju	Netral	Setuju	Sangat Setuju
	**PERUMAHAN**					
Q1	Rumah saya dapat saya akses					
Q2	Rumah saya dapat diakses oleh orang-orang yang datang mengunjungi saya					
	**PARTISIPASI SOSIAL**					
Q3	Ada cukup kesempatan untuk bertemu orang-orang di lingkungan saya					
Q4	Kegiatan dan acara diselenggarakan di tempat-tempat yang dapat diakses oleh saya					
Q5	Informasi tentang kegiatan dan acara sudah cukup untuk saya dan juga cocok untuk saya					
Q6	Saya menemukan berbagai acara dan kegiatan yang cukup bervariasi					
	**RASA HORMAT DAN INKLUSI SOSIAL**					
Q7 *	Terkadang saya mendapatkan komentar yang mengganggu atau negatif karena usia saya					
Q8 *	Saya terkadang mendapatkan diskriminasi karena usia saya					
	**PARTISIPASI SIPIL DAN PEKERJAAN**					
Q9	Saya memiliki kesempatan yang cukup untuk berinteraksi dengan generasi muda					
Q10	Saya merasa seperti anggota masyarakat yang berharga					
	**KOMUNIKASI DAN INFORMASI**					
Q11	Informasi cetak dan digital dari kotamadya dan lembaga sosial lainnya mudah dibaca dalam hal font dan ukuran					
Q12	Informasi cetak dan digital dari kotamadya dan lembaga sosial lainnya ditulis dalam bahasa					

SOESILO
PENERJEMAH TERSUMPAH
B. Indonesia ke B. Inggris - B. Inggris ke B. Indonesia

	yang dapat dimengerti					
	**DUKUNGAN MASYARAKAT DAN LAYANAN KESEHATAN**					
Q13	Suplai perawatan dan kesejahteraan di kota saya sudah cukup bagi saya					
Q14	Ketika saya sakit, saya menerima perawatan dan bantuan yang saya butuhkan					
Q15	Jika perlu, saya dapat dengan mudah menghubungi layanan perawatan dan kesejahteraan melalui telepon dan secara langsung					
Q16	Saya memiliki cukup informasi tentang layanan perawatan dan kesejahteraan di lingkungan saya					
Q17	Pekerja perawatan dan kesejahteraan di lingkungan saya cukup hormat					
	**RUANG DAN BANGUNAN LUAR RUANGAN**					
Q18	Lingkungan saya cukup mudah diakses untuk pejalan kaki beroda atau kursi roda					
Q19	Toko-toko di lingkungan saya cukup mudah diakses dengan alat bantu jalan beroda atau kursi roda					
	**TRANSPORTASI**					
Q20	Saya dapat dengan mudah naik bus atau trem di lingkungan saya					
Q21	Halte bus dan trem di lingkungan saya mudah dijangkau dan digunakan					
	**SITUASI KEUANGAN**					
Q22	Penghasilan saya cukup untuk memenuhi kebutuhan dasar saya tanpa masalah					
Q23	Saya hidup dengan baik dengan penghasilan saya					

AFFIDAVIT

I, SOESILO, a Sworn Translator in the Republic of Indonesia, pursuant to the prevailing laws and regulations in the Republic of Indonesia, hereby certify and declare, in accordance with my oath of office, that this document is a true, faithful, and complete translation from Indonesian to English.

Jakarta, July 08th, 2024

SOESILO

Decree of the Minister of Law and Human Rights R.I.

No. AHU-40 AH.03.07.2022, Reg. No. /SOE/08/07/2024

Jl. Otista III No. 117 Jakarta 13340 email: unggul.translator@gmail.com

The Age-Friendly Cities and Communities Questionnaire AFCCQ (English)

Item	Domain	Totally disagree	Disagree	Neutral	Agree	Totally agree
	**HOUSING**					
Q1	My house is accessible to me					
Q2	My house is accessible to the people who come to visit me					
	**SOCIAL PARTICIPATION**					
Q3	There are enough opportunities to meet people in my neighbourhood					
Q4	Activities and events are organised in places that are accessible to me					
Q5	The information about activities and events is enough for me and also suitable for me					
Q6	I find the range of events and activities sufficiently varied					
	**RESPECT AND SOCIAL INCLUSION**					
Q7*	I sometimes get annoying or negative remarks because of my age					
Q8*	I sometimes face discrimination because of my age					
	**CIVIC PARTICIPATION AND EMPLOYMENT**					
Q9	I have enough opportunities to interact with younger generations					
Q10	I feel like a valued member of society					
	**COMMUNICATION AND INFORMATION**					
Q11	Printed and digital information from the municipality and other social institutions is easy to read in terms of font and size					
Q12	Printed and digital information from the municipality and other social institutions is written in understandable language					
	**COMMUNITY SUPPORT AND HEALTH SERVICES**					
Q13	The supply of care and welfare in my city is enough for me					
Q14	When I am ill, I receive the care and help I need					
Q15	If necessary, I can easily reach care and welfare services by telephone and in person					
Q16	I have enough information about care and welfare services in my neighbourhood					
Q17	Care and welfare workers in my neighbourhood are sufficiently respectful					
	**OUTDOOR SPACES AND BUILDINGS**					
Q18	My neighbourhood is sufficiently accessible for a wheeled walker or wheelchair					
Q19	The shops in my neighbourhood are sufficiently accessible with a wheeled walker or wheelchair					
	**TRANSPORTATION**					
Q20	I can easily get on the bus or tram in my neighbourhood					
Q21	The bus and tram stops in my neighbourhood are easy to reach and use					
	**FINANCIAL SITUATION**					
Q22	My income is sufficient to cover my basic needs without any problems					
Q23	I live well on my income					

The Age-Friendly Cities and Communities Questionnaire AFCCQ (English)

INTERPRETATION AFCCQ TOTAL SCORE AND SEPARATE DOMAINS

	----	---	--	-	+	++	+++	++++
**AFCCQ Total score**	**≤ -35.1**	**-23.1 – -35.0**	**-11.5 – -23.0**	**-11.4 – 0.0**	**0.1 – 11.4**	**11.5 – 23.0**	**23.1 – 35.0**	**≥ 35.1**
Housing	≤ -3.1	-2.1 – -3.0	-1.1 – -2.0	-1.0 – 0.0	0.1 – 1.0	1.1 – 2.0	2.1 – 3.0	≥ 3.1
Social participation	≤ -6.1	-4.1 – -6.0	-2.1 – -4.0	-2.0 – 0.0	0.1 – 2.0	2.1 – 4.0	4.1 – 6.0	≥ 6.1
Respect and social inclusion	≤ -3.1	-2.1 – -3.0	-1.1 – -2.0	-1.0 – 0.0	0.1 – 1.0	1.1 – 2.0	2.1 – 3.0	≥ 3.1
Civic participation and employment	≤ -3.1	-2.1 – -3.0	-1.1 – -2.0	-1.0 – 0.0	0.1 – 1.0	1.1 – 2.0	2.1 – 3.0	≥ 3.1
Communication and information	≤ -3.1	-2.1 – -3.0	-1.1 – -2.0	-1.0 – 0.0	0.1 – 1.0	1.1 – 2.0	2.1 – 3.0	≥ 3.1
Community support and health services	≤ -7.6	-5.1 – -7.5	-2.6 – -5.0	-2.5 – 0.0	0.1 – 2.5	2.6 – 5.0	5.1 – 7.5	≥ 7.6
Outdoor spaces and buildings	≤ -3.1	-2.1 – -3.0	-1.1 – -2.0	-1.0 – 0.0	0.1 – 1.0	1.1 – 2.0	2.1 – 3.0	≥ 3.1
Transportation	≤ -3.1	-2.1 – -3.0	-1.1 – -2.0	-1.0 – 0.0	0.1 – 1.0	1.1 – 2.0	2.1 – 3.0	≥ 3.1
Financial situation	≤ -3.1	-2.1 – -3.0	-1.1 – -2.0	-1.0 – 0.0	0.1 – 1.0	1.1 – 2.0	2.1 – 3.0	≥ 3.1

Scoring system

All questions of the AFCCQ are answered on a 5-point scale, ranging from totally disagree to totally agree.

Scores: -2 = totally disagree; -1 = disagree; 0 = neutral; 1 = agree; 2 = totally agree.

Items marked with an asterisk (*) should be recoded in the opposite direction (-2=2, -1=1, 0=0, 1=-1, 2=-2)

Sum all scores of the AFCCQ for the total score.

Sum all scores of separate domains for the domain specific score.

Reference

Dikken, J., van den Hoven, R.F.M., van Staalduinen, W.H., Hulsebosch-Janssen, L.M.T., van Hoof, J. (2020) How older people experience the age-friendliness of their city: Development of the Age-Friendly Cities and Communities Questionnaire. International Journal of Environmental Research and Public Health 2020;17(18):6867 doi: 10.3390/ijerph17186867

Colophon

This questionnaire is made by J. Dikken (The Hague University of Applied Sciences), R.F.M. van den Hoven (The Hague University of Applied Sciences), W.H. van Staalduinen (AFEdemy), L.M.T. Hulsebosch-Janssen (Hulsebosch Advies) and J. van Hoof (The Hague University of Applied Sciences) with support from the Municipality of The Hague (grant number OCW/2020.1121).

ISBN: 9789083074399
