# Supplementary material for: Assessing age-friendly urban environments in Indonesia: Validation of the Indonesian age-friendly cities and communities questionnaire (AFCCQ) and differences across urban city categories
Source: PLoS One. 2026 Oct 8;21(10):e0358251. doi: 10.1371/journal.pone.0358251 (PMC13648996; doi:10.1371/journal.pone.0358251)
Supplement: Supplementary file 2 [file pone.0358251.s001.docx]

| **Urban Category (*n*, %)** | **City/ District** | **Province** | **Frequency (*n*, %)** |
| --- | --- | --- | --- |
| Small city (12, 2.4%) | Lasem  Sarang  Tegowanu  Sumpiuh  Sendangsari  Pengasih  Pasar Kemis  Bojong Gede  Parung Panjang  Cibinong  Mamuju  Bireuen | Central Java  Central Java  Central Java  Central Java  DI Yogyakarta  DI Yogyakarta  Banten  West Java  West Java  West Java  West Sulawesi  Aceh | 1 (0.2%)  1 (0.2%)  1 (0.2%)  1 (0.2%)  1 (0.2%)  1 (0.2%)  1 (0.2%)  1 (0.2%)  1 (0.2%)  1 (0.2%)  1 (0.2%)  1 (0.2%) |
| Medium-sized city (52, 10.2%) | Purwodadi  Kudus  Cilacap  Purbalingga  Salatiga  Pekalongan  Blitar  Trenggalek  Pasuruan  Kediri  Jember  Madiun  Tuban  Probolinggo  Sidoarjo  Sukabumi  Cianjur  Garut  Tasikmalaya  Pandeglang  Serang Regency  Bangkalan  Sumenep | Central Java  Central Java  Central Java  Central Java  Central Java  Central Java  East Java  East Java  East Java  East Java  East Java  East Java  East Java  East Java  East Java  West Java  West Java  West Java  West Java  Banten  Banten  East Java  East Java | 4 (0.7%)  3 (0.6%)  4 (0.7%)  3 (0.6%)  3 (0.6%)  2 (0.4%)  2 (0.4%)  3 (0.6%)  2 (0.4%)  2 (0.4%)  2 (0.4%)  2 (0.4%)  2 (0.4%)  2 (0.4%)  2 (0.4%)  2 (0.4%)  2 (0.4%)  2 (0.4%)  2 (0.4%)  2 (0.4%)  2 (0.4%)  1 (0.2%)  1 (0.2%) |
| Large city (56, 11.0%) | Bogor  Bekasi Regency  Tangerang Regency  Sleman  Kulon Progo  Purworejo  Rembang  Grobogan  Banyumas  Klaten  Sragen  Ponorogo  Situbondo  Bojonegoro  Tulungagung  Mojokerto  Lampung Timur  Bungo  Banyuasin  Karimun | West Java  West Java  Banten  DI Yogyakarta  DI Yogyakarta  Central Java  Central Java  Central Java  Central Java  Central Java  Central Java  East Java  East Java  East Java  East Java  East Java  Lampung  Jambi  South Sumatra  Riau Islands | 6 (1.3%)  4 (0.7%)  4 (0.7%)  4 (0.7%)  3 (0.6%)  3 (0.6%)  3 (0.6%)  3 (0.6%)  3 (0.6%)  2 (0.4%)  2 (0.4%)  2 (0.4%)  2 (0.4%)  3 (0.6%)  2 (0.4%)  2 (0.4%)  3 (0.6%)  2 (0.4%)  2 (0.4%)  1 (0.2%) |
| Metropolitan city (168, 33.1%) | Bandung  Surabaya  Denpasar  Medan  Palembang  Pekanbaru  Makassar  Pontianak  Yogyakarta  Semarang  Surakarta  Banjarmasin  Balikpapan  Palangka Raya  Bontang  Batu  Lubuklinggau  Bengkulu  Kupang  Manado  Jayapura  Mataram  Palu  Batam  Tegal  Cimahi  Cilegon  Serang | West Java  East Java  Bali  North Sumatra  South Sumatra  Riau  South Sulawesi  West Kalimantan  DI Yogyakarta  Central Java  Central Java  South Kalimantan  East Kalimantan  Central Kalimantan  East Kalimantan  East Java  South Sumatra  Bengkulu  NTT  North Sulawesi  Papua  NTB  Central Sulawesi  Riau Islands  Central Java  West Java  Banten  Banten | 22 (4.3%)  8 (1.6%)  14 (2.9%)  19 (3.8%)  7 (1.4%)  6 (1.3%)  4 (0.7%)  5 (1.0%)  15 (3.0%)  6 (1.3%)  5 (1.0%)  3 (0.6%)  6 (1.3%)  4 (0.7%)  2 (0.4%)  4 (0.7%)  3 (0.6%)  4 (0.7%)  2 (0.4%)  4 (0.7%)  2 (0.4%)  3 (0.6%)  4 (0.7%)  3 (0.6%)  2 (0.4%)  3 (0.6%)  4 (0.7%)  4 (0.7%) |
| Megapolitan city (220, 43.3%) | Jakarta Pusat  Jakarta Selatan  Jakarta Timur  Jakarta Barat  Jakarta Utara  Bekasi  Bogor  Depok  Tangerang  Tangerang Selatan | DKI Jakarta  DKI Jakarta  DKI Jakarta  DKI Jakarta  DKI Jakarta  West Java  West Java  West Java  Banten  Banten | 31 (6.0%)  35 (6.8%)  51 (10.0%)  30 (5.9%)  18 (3.4%)  15 (3.0%)  15 (3.0%)  14 (2.9%)  6 (1.3%)  5 (1.0%) |
