# Supplementary material for: Assessing age-friendly urban environments in Indonesia: Validation of the Indonesian age-friendly cities and communities questionnaire (AFCCQ) and differences across urban city categories
Source: PLoS One. 2026 Oct 8;21(10):e0358251. doi: 10.1371/journal.pone.0358251 (PMC13648996; doi:10.1371/journal.pone.0358251)
Supplement: Supplementary file 3 [file pone.0358251.s004.docx]

12 May 2026

PLOS ONE Editorial Office

plosone@plos.org

**Re: Manuscript ID [PONE-D-25-23142R1]**

Title: Assessing Age-Friendly Urban Environments in Indonesia: Validation of the Indonesian Age-Friendly Cities and Communities Questionnaire (AFCCQ) and Differences Across Urban City Categories

Dear Editor,

Thank you for the opportunity to revise our manuscript and for the guidance from your editorial office. We have addressed all three points and made several additional improvements to strengthen the paper. A point-by-point response follows. Revisions are shown in red in the resubmitted manuscript.

**Editorial office comments**

**Comment 1.** Authors cannot be the sole named individuals responsible for data access.

*Response.* The de-identified dataset, codebook, README, replication script, and data-use agreement have been deposited on Zenodo (DOI: 10.5281/zenodo.20132958) under public access. Metadata are openly visible; the analytic file is released to qualified researchers through Zenodo's restricted-access workflow, which acts as a third-party intermediary independent of the authors. Approved requests receive a time-limited access token issued by Zenodo. Long-term custody of the underlying data rests with the Geriatric Division, Department of Internal Medicine, FKUI–RSCM. Authors are therefore no longer the sole point of data access.

**Comment 2.** Explain restriction in detail, identify who imposed it, and describe long-term storage and access pathway.

*Response.* The restriction was set by the Ethics Committee of FKUI-RSCM (Ref: 24-10-1617; approved 6 January 2025), which limited data sharing to release under a data-use agreement because the combination of age, sex, education, living arrangement, and urban category carries residual re-identification risk for older adults recruited through a single tertiary referral network. The data-use agreement (no re-identification, no redistribution, 12-month access token, citation required) is attached to the Zenodo record. The original dataset is retained for at least ten years on a password-protected institutional server at the Geriatric Division, RSCM.

**Comment 3.** The ethics statement should appear only in the Methods section.

*Response.* The Institutional Review Board Statement and Informed Consent Statement have been removed from the title page. A consolidated ethics paragraph now appears only in the Methods section (Research design and ethics subsection), covering committee name, approval reference, approval date, online and offline consent procedures, and consent by legally authorised representatives for participants unable to read or write. The Abstract, Results, Discussion, and Conclusion no longer mention IRB approval or consent.

**Additional improvements made during revision**

In addition to the editorial points, the following improvements were made to strengthen the manuscript:

**Data integrity.** An internal-consistency check of the source SPSS file identified a clerical inconsistency in the civic-participation subscore column. The civic values in Tables 2 and 5 (previously Table 6) have been corrected by recomputing the subscore directly from items Q9 and Q10. The substantive conclusions are unchanged: the civic domain remains non-significant across city categories.

**Statistical methods.** The Methods now specify Dunn's post-hoc pairwise comparisons with Bonferroni adjustment following significant Kruskal–Wallis tests, and a family-wise Bonferroni correction across the nine domain-level tests. The ICC model is specified as ICC(3,1), single measures, absolute agreement. Interpretation in the Results and Limitations sections has been updated accordingly.

**Content validity.** We now report item-level content validity indices (I-CVI 0.80–1.00) and the scale-level average (S-CVI/Ave = 0.97).

**Citation corrections.** Two citation issues were corrected: the in-text reference to “Omar et al. (2018)” has been changed to “Anggraini et al. (2025)” to match Reference 35, and the citation for the 2010 WHO Global Network has been changed from Reference 8 to Reference 11, which is the appropriate WHO source.

**Overclaiming.** Several sentences in the Discussion and Conclusion that overstated generalisability or implied causal interpretation in a cross-sectional design have been softened.

**Limitations.** Expanded to acknowledge: small-city stratum (n = 12) limiting between-category contrasts, modest variance explained by the single-factor EFA (35.86%) and the need for confirmatory factor analysis, and high financial-domain α (1.00) indicating item redundancy.

**Table numbering.** Tables renumbered consecutively to remove the gap in the original (no Table 4).

**AI disclosure.** A statement on the use of an AI editorial aid has been added to the Acknowledgements, in line with PLOS ONE policy on AI tools.

We are grateful for your careful assessment and look forward to your further consideration.

Sincerely,

**Nina Kemala Sari, MD**

Corresponding author

Geriatric Division, Department of Internal Medicine

Dr. Cipto Mangunkusumo National Referral Hospital

Faculty of Medicine, Universitas Indonesia

nina_geriatri@yahoo.com
