# Supplementary material for: Assessing age-friendly urban environments in Indonesia: Validation of the Indonesian age-friendly cities and communities questionnaire (AFCCQ) and differences across urban city categories
Source: PLoS One. 2026 Oct 8;21(10):e0358251. doi: 10.1371/journal.pone.0358251 (PMC13648996; doi:10.1371/journal.pone.0358251)
Supplement: Supplementary file 4 [file pone.0358251.s005.docx]

**Response to Reviewers**

**Manuscript:** PONE-D-25-23142R1 — Assessing Age-Friendly Urban Environments in Indonesia: Validation of the Indonesian Age-Friendly Cities and Communities Questionnaire (AFCCQ) and Differences Across Urban City Categories

**Journal:** PLOS ONE

We thank the Academic Editor and Reviewer 1 for their careful reading and constructive comments. All reviewer points are addressed below in the order raised. Reviewer comments are shown in italics; our responses follow. Line and section references are to the revised manuscript. A small number of points require confirmation from the author team before resubmission and are marked accordingly; these are listed together at the end of this letter.

**Response to Reviewer 1**

**Introduction**

*Reviewer comment: Please provide more detailed explanations regarding the necessity and importance of assessing age-friendly cities. Furthermore, please emphasize the advantages of the AFCCQ more prominently.*

We agree and have added one focused paragraph to the Introduction (immediately after the paragraph describing the WHO framework). It explains why systematic assessment is necessary in populations that are ageing and urbanising simultaneously; how structured measurement locates barriers specifically in the physical, social, service and transportation environments; and how repeated administration of the same instrument supports monitoring and evaluation rather than one-off description.

**Setting and recruitment**

*Reviewer comment: A potential limitation of this study is the recruitment strategy, which combined both online and offline methods... This potential source of bias should therefore be acknowledged as a limitation in the Discussion section.*

We accept this point and have added a dedicated limitation paragraph. It states that recruitment combined an online channel at a national seniors' expo with offline recruitment at a tertiary referral hospital and a community health centre, and that these channels are likely to select participants differing in literacy, digital access, health status, educational attainment and other socio-demographic characteristics; it notes that such differences may affect the distribution of responses and, through them, the psychometric estimates.

**Participants and sampling**

*Reviewer comment: Please specify the name of the screening test you used to screen for cognitive impairment. Were there any additional inclusion and exclusion criteria? For example, was residence in the city for a specified period of time considered as an inclusion criterion?*

No standardized cognitive screening instrument was used, and we have corrected the Methods to state this plainly rather than implying that a named test was administered. The Methods now read that exclusion for cognitive impairment was based on documented medical history and/or clinical evaluation by the recruiting clinician only, and that no instrument such as the MMSE, MoCA or AMT was administered. We have added the absence of standardized screening to the Limitations, noting that clinical judgement is less sensitive than a validated screening tool and that participants with unrecognised mild cognitive impairment may have been included.

**Translation and cultural adaptation**

*Reviewer comment: Did you obtain permission from the original developers of the instrument to translate and validate it? If so, please state this in the Methods section.*

We have added a paragraph to the Methods describing the licensing position accurately: the AFCCQ is distributed under a Creative Commons Attribution licence via the WHO Age-Friendly World platform, copyright remains with the developers, and users are asked to cite the original development paper, which we do.

On the specific question of permission, we have not asserted that contact with or permission from the development team was obtained, because no documentary evidence of such contact was available when this revision was prepared. The Methods therefore carry an explicit author-confirmation marker at this point. [AUTHOR CONFIRMATION REQUIRED] If correspondence with the AFCCQ development team exists, it should be supplied and the marker replaced with a factual statement; if no such contact occurred, the sentence must be deleted and the omission acknowledged. We have also flagged, for the same reason, that the translation records, back-translation documents, expert-committee minutes and cognitive-debriefing notes underpinning the five-step adaptation procedure were not among the materials available for this revision and should be retained in case the journal requests them.

*Reviewer comment: Please explain why confirmatory factor analysis (CFA) was not performed in this study. Providing a rationale for its omission would help readers better understand the methodological approach.*

We have added a concise rationale to the Limitations. The study was designed as an initial exploratory psychometric evaluation of the Indonesian version, and the available data comprised a single purposive sample. Fitting a confirmatory model to the same sample used to explore the structure would yield optimistically biased fit statistics and would not constitute independent confirmation; no separate validation sample was collected. We state that confirmatory factor analysis, ideally with tests of measurement invariance, is required in an independent and more representative Indonesian sample.

**Results**

*Reviewer comment: The sample was predominantly female, with only 18% of participants being male. Please discuss this gender imbalance as a potential limitation and comment on its possible implications for the psychometric findings.*

The percentage has been updated following the age correction: the eligible sample comprises 412 women (81.6%) and 93 men (18.4%).

We have added a limitation paragraph stating that the reliability and validity estimates reported here therefore describe the performance of the Indonesian AFCCQ primarily among older women, and that their applicability to older men in Indonesia is correspondingly uncertain. We note that measurement invariance by sex was not assessed and that we did not examine whether the factor structure, item–total correlations or internal consistency are stable across male and female subgroups, the male subgroup being too small to support such analyses reliably. In line with the evidence available to us, we make no claim about the direction in which the imbalance may have biased the estimates.

**Discussion**

*Reviewer comment: Although you have compared your findings with previous validation studies, including the original study by Dikken et al., the Discussion would benefit from a more detailed interpretation of the observed differences... discuss the potential reasons for these differences (e.g., cultural context, sample characteristics, or methodological differences).*

We have added one focused comparison paragraph rather than a general expansion of the Discussion. It compares our sample and findings with the original development study by Dikken et al. and with three genuine AFCCQ validation studies — the Turkish validation (Özer et al.), the Romanian study in Bucharest (Ivan et al.), the Polish study in Kraków and Wrocław (Perek-Białas et al.), and the Hebrew validation in Israel (Ayalon et al.).

The paragraph identifies two substantive discrepancies and their plausible explanations. First, respect and social inclusion was the only domain with a negative mean score in our sample, and its two items did not converge with the rest of the scale (Q7 ρ = 0.08, p = 0.086; Q8 ρ = 0.03, p = 0.496); this domain has not been reported as the weakest in the Dutch, Turkish, Romanian or Polish work, and we discuss culturally specific interpretation of respect and inclusion items, and translation wording, as candidate explanations. Second, our exploratory analysis did not recover the nine-domain structure, whereas the development work established it on an independent and more representative sample using a different analytical route. We discuss recruitment, age and sex composition, and the extraction and retention procedures as plausible contributors, while stating candidly that the present design cannot distinguish between them.

*Reviewer comment: Dikken et al. have published specific guidelines for the translation and psychometric evaluation of this instrument. Please refer to these guidelines in the Discussion section and discuss your findings in light of the recommendations provided by the original developers.*

We have addressed this in the same comparison paragraph and in the Methods. The Discussion now notes that the AFCCQ was developed through a COSMIN-based procedure that reduced an initial pool of 64 candidate questions to the final 23 items, that the developers make a validation manual available on request, and that new language versions are expected to follow the COSMIN protocol and to involve the development team at the translation stage. We state that future Indonesian work should be conducted explicitly against that guidance.

We have been careful not to overstate our own conformity with those recommendations. Our adaptation followed a five-step forward-translation, reconciliation, blinded back-translation, expert-review and cognitive-debriefing procedure as described in the Methods, but as noted above the supporting documentation was not available for this revision and is flagged for author confirmation.

**Response to the Journal Requirements**

Reference list. We have reviewed the full reference list. We found no retracted or materially corrected references. We made four evidence-based changes, each explained in the claim-and-reference audit accompanying this letter (S4 File):

- Reference 1 no longer supports the opening sentence, because we corrected the global ageing figures. The previous text stated that approximately 720 million people are aged 65 or older and that one in five people worldwide will be older adults by 2030, which mixed the 65+ and 60+ thresholds. The text now uses the WHO figures directly (1 billion people aged 60+ in 2020 rising to 1.4 billion by 2030; one in six people aged 60 or over by 2030), cited to the WHO Ageing and Health fact sheet.
- Reference 27 concerned the Pearson product-moment correlation coefficient but was cited to support a Spearman-based item–total procedure. It has been replaced with de Winter, Gosling and Potter (2016), which directly compares the Pearson and Spearman coefficients.
- Reference 33 concerned the Age-Friendly Environment Assessment Tool, a different instrument, and was cited in support of a statement about cross-cultural validation of the AFCCQ. The sentence has been deleted as peripheral, and reference 33 now cites the Hebrew AFCCQ validation, which is used in the new comparison paragraph.
- References 8, 21 and 41 had incomplete or inaccurate metadata (missing article numbers, an abbreviated author name). These have been corrected and DOIs added, verified against the official AFCCQ page and the publishers' records.

Data availability. The Data Availability Statement has been revised to describe the deposited files accurately, including the fact that geographic information is released only as the five-level urban category, so the city-level counts in S1 Table cannot be regenerated from the public file.

Supporting information. The previous manuscript labelled the Indonesian questionnaire as “S1 File”, but the supplementary file actually supplied is a city/location table. The labels have been corrected: S1 Table is the city distribution table, S2 File is the Indonesian AFCCQ instrument, S3 File is the analysis script and log, and S4 File is the claim and reference audit. [AUTHOR CONFIRMATION REQUIRED] The Indonesian instrument file was not among the materials available for this revision and must be attached before resubmission, or the S2 File reference deleted.

Generative AI disclosure. Our previous statement said that AI was not used to conduct statistical analyses. That statement is no longer accurate for this revision and we have replaced it rather than leave it standing. The revised disclosure states that a large language model was used to write the reproducible analysis script that recomputed the results after the eligibility error was identified, that the authors specified the analysis plan, executed and inspected the code, and independently verified every reported value against the source data and against SPSS output, and that the authors take full responsibility for the integrity and accuracy of all reported results.

Other internal corrections. “Table 54” has been corrected to Table 4 and table and figure numbering checked for sequence. The discrepancy between the 84 municipalities stated in the text and the 93 location labels listed in the supplement has been resolved and explained in S1 Table. Nominal p-values are no longer described as “significant” anywhere in the manuscript, and the Conclusion has been tempered so that it no longer asserts city-category differences that did not survive correction for multiplicity.

We are grateful to the reviewer for comments that led us to re-examine the analytic dataset, and we hope the revised manuscript is now suitable for publication.
