# Supplementary material for: Assessing age-friendly urban environments in Indonesia: Validation of the Indonesian age-friendly cities and communities questionnaire (AFCCQ) and differences across urban city categories
Source: PLoS One. 2026 Oct 8;21(10):e0358251. doi: 10.1371/journal.pone.0358251 (PMC13648996; doi:10.1371/journal.pone.0358251)
Supplement: Peer review history

# Peer review: Decision Letter 0

**DOI:** 10.1371/journal.pone.0358251.r001

**Authors:** Mohammad Saadati

5 Oct 2025

Dear Dr. Sari,

Thank you for submitting your manuscript to PLOS ONE. After careful consideration, we feel that it has merit but does not fully meet PLOS ONE’s publication criteria as it currently stands. Therefore, we invite you to submit a revised version of the manuscript that addresses the points raised during the review process.

Please submit your revised manuscript by Nov 19 2025 11:59PM. If you will need more time than this to complete your revisions, please reply to this message or contact the journal office at plosone@plos.org. When you’re ready to submit your revision, log on to https://www.editorialmanager.com/pone/ and select the 'Submissions Needing Revision' folder to locate your manuscript file.

- A rebuttal letter that responds to each point raised by the academic editor and reviewer(s). You should upload this letter as a separate file labeled 'Response to Reviewers'.
- A marked-up copy of your manuscript that highlights changes made to the original version. You should upload this as a separate file labeled 'Revised Manuscript with Track Changes'.
- An unmarked version of your revised paper without tracked changes. You should upload this as a separate file labeled 'Manuscript'.

If you would like to make changes to your financial disclosure, please include your updated statement in your cover letter. Guidelines for resubmitting your figure files are available below the reviewer comments at the end of this letter.

If applicable, we recommend that you deposit your laboratory protocols in protocols.io to enhance the reproducibility of your results. Protocols.io assigns your protocol its own identifier (DOI) so that it can be cited independently in the future. For instructions see: https://journals.plos.org/plosone/s/submission-guidelines#loc-laboratory-protocols. Additionally, PLOS ONE offers an option for publishing peer-reviewed Lab Protocol articles, which describe protocols hosted on protocols.io. Read more information on sharing protocols at https://plos.org/protocols?utm_medium=editorial-email&utm_source=authorletters&utm_campaign=protocols.

We look forward to receiving your revised manuscript.

Kind regards,

Mohammad Saadati

Academic Editor

PLOS ONE

Journal Requirements:

When submitting your revision, we need you to address these additional requirements.

1.Please ensure that your manuscript meets PLOS ONE's style requirements, including those for file naming. The PLOS ONE style templates can be found at

https://journals.plos.org/plosone/s/file?id=wjVg/PLOSOne_formatting_sample_main_body.pdf and

https://journals.plos.org/plosone/s/file?id=ba62/PLOSOne_formatting_sample_title_authors_affiliations.pdf

2. Thank you for stating the following financial disclosure:

“This study was funded by PUTI Q1 2022 Batch 1 (Grant Number: NKB-426/UN2.RST/HKP.05.00/2022) by Universitas Indonesia.”

Please state what role the funders took in the study.  If the funders had no role, please state: "The funders had no role in study design, data collection and analysis, decision to publish, or preparation of the manuscript."

If this statement is not correct you must amend it as needed.

Please include this amended Role of Funder statement in your cover letter; we will change the online submission form on your behalf.

3. Please note that funding information should not appear in any section or other areas of your manuscript. We will only publish funding information present in the Funding Statement section of the online submission form. Please remove any funding-related text from the manuscript.

4. In the online submission form, you indicated that “The data supporting this study's findings are available from the corresponding author upon reasonable request.”

All PLOS journals now require all data underlying the findings described in their manuscript to be freely available to other researchers, either 1. In a public repository, 2. Within the manuscript itself, or 3. Uploaded as supplementary information.

This policy applies to all data except where public deposition would breach compliance with the protocol approved by your research ethics board. If your data cannot be made publicly available for ethical or legal reasons (e.g., public availability would compromise patient privacy), please explain your reasons on resubmission and your exemption request will be escalated for approval.

5. When completing the data availability statement of the submission form, you indicated that you will make your data available on acceptance. We strongly recommend all authors decide on a data sharing plan before acceptance, as the process can be lengthy and hold up publication timelines. Please note that, though access restrictions are acceptable now, your entire data will need to be made freely accessible if your manuscript is accepted for publication. This policy applies to all data except where public deposition would breach compliance with the protocol approved by your research ethics board. If you are unable to adhere to our open data policy, please kindly revise your statement to explain your reasoning and we will seek the editor's input on an exemption. Please be assured that, once you have provided your new statement, the assessment of your exemption will not hold up the peer review process.

6. Thank you for stating the following financial disclosure:

“This study was funded by PUTI Q1 2022 Batch 1 (Grant Number: NKB-426/UN2.RST/HKP.05.00/2022) by Universitas Indonesia.”

Please state what role the funders took in the study.  If the funders had no role, please state: "The funders had no role in study design, data collection and analysis, decision to publish, or preparation of the manuscript."

If this statement is not correct you must amend it as needed.

Please include this amended Role of Funder statement in your cover letter; we will change the online submission form on your behalf.

7. We note that the grant information you provided in the ‘Funding Information’ and ‘Financial Disclosure’ sections do not match.

When you resubmit, please ensure that you provide the correct grant numbers for the awards you received for your study in the ‘Funding Information’ section.

8. Your ethics statement should only appear in the Methods section of your manuscript. If your ethics statement is written in any section besides the Methods, please delete it from any other section.

9. Please include captions for your Supporting Information files at the end of your manuscript, and update any in-text citations to match accordingly. Please see our Supporting Information guidelines for more information: http://journals.plos.org/plosone/s/supporting-information.

10. If the reviewer comments include a recommendation to cite specific previously published works, please review and evaluate these publications to determine whether they are relevant and should be cited. There is no requirement to cite these works unless the editor has indicated otherwise.

[Note: HTML markup is below. Please do not edit.]

Reviewer's Responses to Questions

**Comments to the Author**

1. Is the manuscript technically sound, and do the data support the conclusions?

Reviewer #1: Partly

Reviewer #2: Partly

**********

2. Has the statistical analysis been performed appropriately and rigorously?

Reviewer #1: No

Reviewer #2: Yes

**********

3. Have the authors made all data underlying the findings in their manuscript fully available?

The PLOS Data policy

Reviewer #1: Yes

Reviewer #2: No

**********

4. Is the manuscript presented in an intelligible fashion and written in standard English?

Reviewer #1: No

Reviewer #2: Yes

**********

Reviewer #1: Dear Editor,

Thank you for the opportunity to review the manuscript, "[ Evaluating Age-Friendliness of Indonesian Urban Environments: Validation of the Age Friendly Cities and Communities Questionnaire (AFCCQ) and Its Implications for Older Adults]" for PLOS ONE. The topic of for age-friendly city assessment tools is highly relevant and important, and I appreciate the authors' efforts in this area.

Overall, while the topic is pertinent, the methodological approach described in the paper presents significant flaws that prevent it from fully meeting the established criteria for a rigorous psychometric study. Specifically, there is a critical absence of information regarding the assessment of both face validity and content validity, which are fundamental initial steps in the psychometric evaluation of any instrument.

Comments for Authors

I appreciate the authors’ efforts to evaluate psychometrics properties of Age Friendly Cities and Communities Questionnaire (AFCCQ), an area of growing relevance to urban planning and public health. The topic is timely and the initiative is commendable. Upon reviewing the methodology, however, I noticed several areas that may warrant further clarification or reconsideration to strengthen the psychometric rigor of the study. Here are my main comments and suggestions for improvement.

-The English language quality in the manuscript is suboptimal and needs substantial improvement. I strongly recommend a thorough review by a native English speaker or professional editing service

-To ensure the language in the manuscript aligns with current best practices in gerontology and avoids ageist terminology, please replace terms like "old" , “elderly” and other similar expressions that can carry negative connotations or promote an anti-aging perspective. Focusing on precise, respectful, and person-first language (e.g., "older adults," "older people," "aging individuals") will significantly enhance the paper's inclusivity and scholarly tone.

Introduction

-To further strengthen the introduction's relevance to an age-friendly cities concept, it might be more impactful to address the unique challenges associated with urbanization and urban living for older adults.

-To further strengthen the paper, consider referencing other existing age-friendly city assessment tools and clearly articulating the specific advantages of the AFCCQ. For instance, highlighting that the AFCCQ is one of the few tools assessing age-friendliness from the direct perspective of older adults would significantly underscore its unique value and contribution.

Materials and Methods

-The methods section is written quite superficially and doesn't fully reflect the rigorous methodology required for psychometric studies. To address this, I suggest a comprehensive revision to include a more in-depth description of each methodological step taken.

-In this study, only Exploratory Factor Analysis (EFA) for construct validity and reliability were evaluated. The current methods section lacks any mention of how content or face validity were established using for example CVR and CVI. These are crucial components of psychometric evaluation. This should also include following the instrument designers' instructions for the psychometric testing of the AFCCQ.

-The sampling method is very vague. It is only mentioned in the method section that offline and online methods were used to collect data? Through which application? Which datasource? The authors said: “Offline data collection took place at the Paris Community Health Center and Dr. Cipto Mangunkusumo National Referral Hospital, both located in Jakarta.” What are “Paris Community Health Center” and “Dr. Cipto Mangunkusumo National Referral Hospital”? Are they databases that contain information on all older people in Jakarta? It is important to clarify the specific database(s) from which this critical demographic information was sourced. This detail adds significant weight to the contextual background of your work.

-The sampling method appears to have potentially exposed the study to selection bias. Purposive sampling through online and offline methods questions the ability to reach all older adults in the target population. The unbalanced number of male and female participants may be a reflection of this issue.

- In the case of illiterate and low-literate elderly people, who completed the questionnaire? Did the researcher complete the questionnaire?

-One of the goals of AFCCQ is to enable comparisons between cities and communities regarding age-friendliness. Please specify the names of the cities surveyed and provide a brief explanation of their key geographic and demographic characteristics. Simply referring to " Small city, Medium-sized city, Large city, Metropolitan city" is insufficient; detailing these characteristics will significantly enhance the depth and validity of any comparisons.

Discussion

-The discussion section is also currently quite simple and superficial, lacking the depth expected for a comprehensive interpretation of your findings. A more thorough discussion is needed to fully contextualize your results within the existing literature and to explore their broader implications. How do your findings align with or diverge from previous psychometric studies of this instrument or similar instruments?

-The points discussed are not your study limitation. Examining the age-friendliness of communities from the perspective of seniors is not the limit of your study. For this section to be most impactful, I suggest revising it to highlight the inherent constraints and potential weaknesses of your study's specific methodology, sample, or analyses that might impact the generalizability or interpretation of your findings.

Reviewer #2: Dear Editor

Thanks for the opportunity to review this paper. This is a valuable and timely study that addresses the important topic of age-friendliness in Indonesian urban environments. The validation of the Age-Friendly Cities and Communities Questionnaire (AFCCQ) for the Indonesian context is a significant contribution to the field, providing a crucial tool for researchers, urban planners, and policymakers. The manuscript is well-structured, clearly written, and employs a robust methodology to assess the instrument's validity and reliability. The findings offer practical insights into the specific domains—such as housing, social participation, and transportation—that require attention to improve the quality of life for older adults in Indonesia. While the study is methodologically sound, there are a few critical areas that require clarification to ensure the manuscript's integrity and reproducibility as following:

- There are some inconsistent information along the manuscript which should be resolved:

-Data collection period was stated to be from September to October 2024 in the system abstract while it is different in the manuscript abstract and text.

-Ethical approval code is also different in the statement which is uploaded to the submission system and in the methods section.

-Correlation analysis method is declared to be Pearson product-moment correlation but the discussion section refers to spearman correlation analysis. Please ensure the correct test is reported consistently. Given the ordinal nature of Likert-scale data, Spearman's correlation may be more appropriate, but whichever is used should be stated consistently.

-Some items needs to be clarified:

-Authors declared in the results section that single-factor model was maintained based on the initial eigenvalue of 8.25 and the scree plot. However, Table 4 is presented as a rotated component matrix showing at least seven different components with item loading onto separate factors. The authors should clarify whether a single-factor or multi-factor solution was adopted and update the results and discussion section based on this.

-In the Methods section (Page 9), the list of the nine AFCCQ domains contains repetitions. "Outdoor spaces and buildings" and "transportation" are each listed twice. Please correct this.

-In the reliability analysis, the "Financial situation" domain is reported to have a Cronbach's alpha of 1.00. While mathematically possible, a perfect alpha is highly unusual and can suggest item redundancy. A brief comment on this perfect correlation in the Discussion section would be insightful.

-Table 1 ‘The socioeconomic and demographic characteristics of study participants’ doesn’t include the frequency of participants from ‘Megapolitan cities’.

-The text identifies the sampling method as ‘purposive’. Please provide more details on the recruitment process and also method of participants approaching during online senior expo event and at the offline health centres to minimize selection bias? How written consent was approached in online data collection?

-Please clarify that was the first 100 participants who were recruited for tool reliability check, out of the final 508 participants?

-Please provide the mean score of age-friendliness for each category of cities in the Table 6 along with their comparison.

-The manuscript is well-written, but a final proofread is recommended to catch minor typos.

Bests

**********

what does this mean?). If published, this will include your full peer review and any attached files.

If you choose “no”, your identity will remain anonymous but your review may still be made public.

**Do you want your identity to be public for this peer review?**  For information about this choice, including consent withdrawal, please see our Privacy Policy

Reviewer #1: No

Reviewer #2: No

**********

[NOTE: If reviewer comments were submitted as an attachment file, they will be attached to this email and accessible via the submission site. Please log into your account, locate the manuscript record, and check for the action link "View Attachments". If this link does not appear, there are no attachment files.]

While revising your submission, please upload your figure files to the Preflight Analysis and Conversion Engine (PACE) digital diagnostic tool, https://pacev2.apexcovantage.com/. PACE helps ensure that figures meet PLOS requirements. To use PACE, you must first register as a user. Registration is free. Then, login and navigate to the UPLOAD tab, where you will find detailed instructions on how to use the tool. If you encounter any issues or have any questions when using PACE, please email PLOS at figures@plos.org. Please note that Supporting Information files do not need this step.

# Author comment: Author response to Decision Letter 1

**DOI:** 10.1371/journal.pone.0358251.r002

12 May 2026

12 May 2026

PLOS ONE Editorial Office

plosone@plos.org

Re: Manuscript ID [PONE-D-25-23142R1]

Title: Assessing Age-Friendly Urban Environments in Indonesia: Validation of the Indonesian Age-Friendly Cities and Communities Questionnaire (AFCCQ) and Differences Across Urban City Categories

Dear Editor,

Thank you for the opportunity to revise our manuscript and for the guidance from your editorial office. We have addressed all three points and made several additional improvements to strengthen the paper. A point-by-point response follows. Revisions are shown in red in the resubmitted manuscript.

Editorial office comments

Comment 1. Authors cannot be the sole named individuals responsible for data access.

Response. The de-identified dataset, codebook, README, replication script, and data-use agreement have been deposited on Zenodo (DOI: 10.5281/zenodo.20132958) under public access. Metadata are openly visible; the analytic file is released to qualified researchers through Zenodo's restricted-access workflow, which acts as a third-party intermediary independent of the authors. Approved requests receive a time-limited access token issued by Zenodo. Long-term custody of the underlying data rests with the Geriatric Division, Department of Internal Medicine, FKUI–RSCM. Authors are therefore no longer the sole point of data access.

Comment 2. Explain restriction in detail, identify who imposed it, and describe long-term storage and access pathway.

Response. The restriction was set by the Ethics Committee of FKUI-RSCM (Ref: 24-10-1617; approved 6 January 2025), which limited data sharing to release under a data-use agreement because the combination of age, sex, education, living arrangement, and urban category carries residual re-identification risk for older adults recruited through a single tertiary referral network. The data-use agreement (no re-identification, no redistribution, 12-month access token, citation required) is attached to the Zenodo record. The original dataset is retained for at least ten years on a password-protected institutional server at the Geriatric Division, RSCM.

Comment 3. The ethics statement should appear only in the Methods section.

Response. The Institutional Review Board Statement and Informed Consent Statement have been removed from the title page. A consolidated ethics paragraph now appears only in the Methods section (Research design and ethics subsection), covering committee name, approval reference, approval date, online and offline consent procedures, and consent by legally authorised representatives for participants unable to read or write. The Abstract, Results, Discussion, and Conclusion no longer mention IRB approval or consent.

Additional improvements made during revision

In addition to the editorial points, the following improvements were made to strengthen the manuscript:

Data integrity. An internal-consistency check of the source SPSS file identified a clerical inconsistency in the civic-participation subscore column. The civic values in Tables 2 and 5 (previously Table 6) have been corrected by recomputing the subscore directly from items Q9 and Q10. The substantive conclusions are unchanged: the civic domain remains non-significant across city categories.

Statistical methods. The Methods now specify Dunn's post-hoc pairwise comparisons with Bonferroni adjustment following significant Kruskal–Wallis tests, and a family-wise Bonferroni correction across the nine domain-level tests. The ICC model is specified as ICC(3,1), single measures, absolute agreement. Interpretation in the Results and Limitations sections has been updated accordingly.

Content validity. We now report item-level content validity indices (I-CVI 0.80–1.00) and the scale-level average (S-CVI/Ave = 0.97).

Citation corrections. Two citation issues were corrected: the in-text reference to “Omar et al. (2018)” has been changed to “Anggraini et al. (2025)” to match Reference 35, and the citation for the 2010 WHO Global Network has been changed from Reference 8 to Reference 11, which is the appropriate WHO source.

Overclaiming. Several sentences in the Discussion and Conclusion that overstated generalisability or implied causal interpretation in a cross-sectional design have been softened.

Limitations. Expanded to acknowledge: small-city stratum (n = 12) limiting between-category contrasts, modest variance explained by the single-factor EFA (35.86%) and the need for confirmatory factor analysis, and high financial-domain α (1.00) indicating item redundancy.

Table numbering. Tables renumbered consecutively to remove the gap in the original (no Table 4).

AI disclosure. A statement on the use of an AI editorial aid has been added to the Acknowledgements, in line with PLOS ONE policy on AI tools.

We are grateful for your careful assessment and look forward to your further consideration.

Sincerely,

Nina Kemala Sari, MD

Corresponding author

Geriatric Division, Department of Internal Medicine

Dr. Cipto Mangunkusumo National Referral Hospital

Faculty of Medicine, Universitas Indonesia

nina_geriatri@yahoo.com

Attachment Submitted filename: AFCCQ Rebuttal Letter.docx

# Peer review: Decision Letter 1

**DOI:** 10.1371/journal.pone.0358251.r003

**Authors:** Mohammad Saadati

12 Jul 2026

Dear Dr. Sari,

Thank you for submitting your manuscript to PLOS ONE. After careful consideration, we feel that it has merit but does not fully meet PLOS ONE’s publication criteria as it currently stands. Therefore, we invite you to submit a revised version of the manuscript that addresses the points raised during the review process.

Please submit your revised manuscript by Aug 26 2026 11:59PM. If you will need more time than this to complete your revisions, please reply to this message or contact the journal office at plosone@plos.org. When you're ready to submit your revision, log on to https://www.editorialmanager.com/pone/ and select the 'Submissions Needing Revision' folder to locate your manuscript file.

- A letter that responds to each point raised by the academic editor and reviewer(s). You should upload this letter as a separate file labeled 'Response to Reviewers'.
- A marked-up copy of your manuscript that highlights changes made to the original version. You should upload this as a separate file labeled 'Revised Manuscript with Track Changes'.
- An unmarked version of your revised paper without tracked changes. You should upload this as a separate file labeled 'Manuscript'.

If you would like to make changes to your financial disclosure, please include your updated statement in your cover letter. Guidelines for resubmitting your figure files are available below the reviewer comments at the end of this letter.

If applicable, we recommend that you deposit your laboratory protocols in protocols.io to enhance the reproducibility of your results. Protocols.io assigns your protocol its own identifier (DOI) so that it can be cited independently in the future. For instructions see: https://journals.plos.org/plosone/s/submission-guidelines#loc-laboratory-protocols. Additionally, PLOS ONE offers an option for publishing peer-reviewed Lab Protocol articles, which describe protocols hosted on protocols.io. Read more information on sharing protocols at https://plos.org/protocols?utm_medium=editorial-email&utm_source=authorletters&utm_campaign=protocols.

As the corresponding author, your ORCID iD is verified in the submission system and will appear in the published article. PLOS supports the use of ORCID, and we encourage all coauthors to register for an ORCID iD and use it as well. Please encourage your coauthors to verify their ORCID iD within the submission system before final acceptance, as unverified ORCID iDs will not appear in the published article. *Only*  the individual author can complete the verification step; PLOS staff *cannot*  verify ORCID iDs on behalf of authors.

We look forward to receiving your revised manuscript.

Kind regards,

Mohammad Saadati

Academic Editor

PLOS One

Journal Requirements:

If the reviewer comments include a recommendation to cite specific previously published works, please review and evaluate these publications to determine whether they are relevant and should be cited. There is no requirement to cite these works unless the editor has indicated otherwise.

Please review your reference list to ensure that it is complete and correct. If you have cited papers that have been retracted, please include the rationale for doing so in the manuscript text, or remove these references and replace them with relevant current references. Any changes to the reference list should be mentioned in the rebuttal letter that accompanies your revised manuscript. If you need to cite a retracted article, indicate the article’s retracted status in the References list and also include a citation and full reference for the retraction notice.

[Note: HTML markup is below. Please do not edit.]

Reviewers' comments:

Reviewer's Responses to Questions

**Comments to the Author**

Reviewer #1: (No Response)

**********

2. Is the manuscript technically sound, and do the data support the conclusions?

Reviewer #1: (No Response)

**********

3. Has the statistical analysis been performed appropriately and rigorously?

Reviewer #1: (No Response)

**********

4. Have the authors made all data underlying the findings in their manuscript fully available?

The PLOS Data policy

Reviewer #1: (No Response)

**********

5. Is the manuscript presented in an intelligible fashion and written in standard English?

Reviewer #1: (No Response)

**********

Reviewer #1: I would like to thank the authors for their efforts in conducting this study. Overall, the manuscript is well written and addresses an important topic. The comments provided above are intended to strengthen the manuscript and improve its clarity and methodological rigor.

INTRUCUTION

Please provide more detailed explanations regarding the necessity and importance of assessing age-friendly cities. Furthermore, please emphasize the advantages of the AFCCQ more prominently.

Setting and recruitment

A potential limitation of this study is the recruitment strategy, which combined both online and offline methods. Although generalizability is not the primary concern in psychometric validation studies, the use of different recruitment approaches may have introduced selection bias. For example, participants recruited through the expo may differ from those recruited in hospital settings with respect to literacy, digital access, or other socio-demographic characteristics. Such differences could have influenced participants' responses and, consequently, the observed psychometric properties of the instrument. This potential source of bias should therefore be acknowledged as a limitation in the Discussion section.

Participants and sampling

"Individuals with confirmed cognitive impairment, based on medical history or clinical evaluation, were excluded. Please specify the name of the screening test you used to screen for cognitive impairment.

Were there any additional inclusion and exclusion criteria? For example, was residence in the city for a specified period of time considered as an inclusion criterion?

Translation and cultural adaptation

Did you obtain permission from the original developers of the instrument to translate and validate it? If so, please state this in the Methods section.

Please explain why confirmatory factor analysis (CFA) was not performed in this study. Providing a rationale for its omission would help readers better understand the methodological approach.

Results

The sample was predominantly female, with only 18% of participants being male. Please discuss this gender imbalance as a potential limitation and comment on its possible implications for the psychometric findings.

Discussion

Although you have compared your findings with previous validation studies, including the original study by Dikken et al., the Discussion would benefit from a more detailed interpretation of the observed differences. In particular, please highlight any discrepancies between your findings and those of previous studies and discuss the potential reasons for these differences (e.g., cultural context, sample characteristics, or methodological differences).

Dikken et al. have published specific guidelines for the translation and psychometric evaluation of this instrument. Please refer to these guidelines in the Discussion section and discuss your findings in light of the recommendations provided by the original developers.

**********

what does this mean?). If published, this will include your full peer review and any attached files.

If you choose “no”, your identity will remain anonymous but your review may still be made public.

**Do you want your identity to be public for this peer review?**  For information about this choice, including consent withdrawal, please see our Privacy Policy

Reviewer #1: No

**********

[NOTE: If reviewer comments were submitted as an attachment file, they will be attached to this email and accessible via the submission site. Please log into your account, locate the manuscript record, and check for the action link "View Attachments". If this link does not appear, there are no attachment files.]

To ensure your figures meet our technical requirements, please review our figure guidelines: https://journals.plos.org/plosone/s/figures

You may also use PLOS’s free figure tool, NAAS, to help you prepare publication quality figures: https://journals.plos.org/plosone/s/figures#loc-tools-for-figure-preparation.

NAAS will assess whether your figures meet our technical requirements by comparing each figure against our figure specifications.

# Author comment: Author response to Decision Letter 2

**DOI:** 10.1371/journal.pone.0358251.r004

26 Aug 2026

Response to Reviewers

Manuscript: PONE-D-25-23142R1 — Assessing Age-Friendly Urban Environments in Indonesia: Validation of the Indonesian Age-Friendly Cities and Communities Questionnaire (AFCCQ) and Differences Across Urban City Categories

Journal: PLOS ONE

We thank the Academic Editor and Reviewer 1 for their careful reading and constructive comments. All reviewer points are addressed below in the order raised. Reviewer comments are shown in italics; our responses follow. Line and section references are to the revised manuscript. A small number of points require confirmation from the author team before resubmission and are marked accordingly; these are listed together at the end of this letter.

Response to Reviewer 1

Introduction

Reviewer comment: Please provide more detailed explanations regarding the necessity and importance of assessing age-friendly cities. Furthermore, please emphasize the advantages of the AFCCQ more prominently.

We agree and have added one focused paragraph to the Introduction (immediately after the paragraph describing the WHO framework). It explains why systematic assessment is necessary in populations that are ageing and urbanising simultaneously; how structured measurement locates barriers specifically in the physical, social, service and transportation environments; and how repeated administration of the same instrument supports monitoring and evaluation rather than one-off description.

Setting and recruitment

Reviewer comment: A potential limitation of this study is the recruitment strategy, which combined both online and offline methods... This potential source of bias should therefore be acknowledged as a limitation in the Discussion section.

We accept this point and have added a dedicated limitation paragraph. It states that recruitment combined an online channel at a national seniors' expo with offline recruitment at a tertiary referral hospital and a community health centre, and that these channels are likely to select participants differing in literacy, digital access, health status, educational attainment and other socio-demographic characteristics; it notes that such differences may affect the distribution of responses and, through them, the psychometric estimates.

Participants and sampling

Reviewer comment: Please specify the name of the screening test you used to screen for cognitive impairment. Were there any additional inclusion and exclusion criteria? For example, was residence in the city for a specified period of time considered as an inclusion criterion?

No standardized cognitive screening instrument was used, and we have corrected the Methods to state this plainly rather than implying that a named test was administered. The Methods now read that exclusion for cognitive impairment was based on documented medical history and/or clinical evaluation by the recruiting clinician only, and that no instrument such as the MMSE, MoCA or AMT was administered. We have added the absence of standardized screening to the Limitations, noting that clinical judgement is less sensitive than a validated screening tool and that participants with unrecognised mild cognitive impairment may have been included.

Translation and cultural adaptation

Reviewer comment: Did you obtain permission from the original developers of the instrument to translate and validate it? If so, please state this in the Methods section.

We have added a paragraph to the Methods describing the licensing position accurately: the AFCCQ is distributed under a Creative Commons Attribution licence via the WHO Age-Friendly World platform, copyright remains with the developers, and users are asked to cite the original development paper, which we do.

On the specific question of permission, we have not asserted that contact with or permission from the development team was obtained, because no documentary evidence of such contact was available when this revision was prepared. The Methods therefore carry an explicit author-confirmation marker at this point. [AUTHOR CONFIRMATION REQUIRED] If correspondence with the AFCCQ development team exists, it should be supplied and the marker replaced with a factual statement; if no such contact occurred, the sentence must be deleted and the omission acknowledged. We have also flagged, for the same reason, that the translation records, back-translation documents, expert-committee minutes and cognitive-debriefing notes underpinning the five-step adaptation procedure were not among the materials available for this revision and should be retained in case the journal requests them.

Reviewer comment: Please explain why confirmatory factor analysis (CFA) was not performed in this study. Providing a rationale for its omission would help readers better understand the methodological approach.

We have added a concise rationale to the Limitations. The study was designed as an initial exploratory psychometric evaluation of the Indonesian version, and the available data comprised a single purposive sample. Fitting a confirmatory model to the same sample used to explore the structure would yield optimistically biased fit statistics and would not constitute independent confirmation; no separate validation sample was collected. We state that confirmatory factor analysis, ideally with tests of measurement invariance, is required in an independent and more representative Indonesian sample.

Results

Reviewer comment: The sample was predominantly female, with only 18% of participants being male. Please discuss this gender imbalance as a potential limitation and comment on its possible implications for the psychometric findings.

The percentage has been updated following the age correction: the eligible sample comprises 412 women (81.6%) and 93 men (18.4%).

We have added a limitation paragraph stating that the reliability and validity estimates reported here therefore describe the performance of the Indonesian AFCCQ primarily among older women, and that their applicability to older men in Indonesia is correspondingly uncertain. We note that measurement invariance by sex was not assessed and that we did not examine whether the factor structure, item–total correlations or internal consistency are stable across male and female subgroups, the male subgroup being too small to support such analyses reliably. In line with the evidence available to us, we make no claim about the direction in which the imbalance may have biased the estimates.

Discussion

Reviewer comment: Although you have compared your findings with previous validation studies, including the original study by Dikken et al., the Discussion would benefit from a more detailed interpretation of the observed differences... discuss the potential reasons for these differences (e.g., cultural context, sample characteristics, or methodological differences).

We have added one focused comparison paragraph rather than a general expansion of the Discussion. It compares our sample and findings with the original development study by Dikken et al. and with three genuine AFCCQ validation studies — the Turkish validation (Özer et al.), the Romanian study in Bucharest (Ivan et al.), the Polish study in Kraków and Wrocław (Perek-Białas et al.), and the Hebrew validation in Israel (Ayalon et al.).

The paragraph identifies two substantive discrepancies and their plausible explanations. First, respect and social inclusion was the only domain with a negative mean score in our sample, and its two items did not converge with the rest of the scale (Q7 ρ = 0.08, p = 0.086; Q8 ρ = 0.03, p = 0.496); this domain has not been reported as the weakest in the Dutch, Turkish, Romanian or Polish work, and we discuss culturally specific interpretation of respect and inclusion items, and translation wording, as candidate explanations. Second, our exploratory analysis did not recover the nine-domain structure, whereas the development work established it on an independent and more representative sample using a different analytical route. We discuss recruitment, age and sex composition, and the extraction and retention procedures as plausible contributors, while stating candidly that the present design cannot distinguish between them.

Reviewer comment: Dikken et al. have published specific guidelines for the translation and psychometric evaluation of this instrument. Please refer to these guidelines in the Discussion section and discuss your findings in light of the recommendations provided by the original developers.

We have addressed this in the same comparison paragraph and in the Methods. The Discussion now notes that the AFCCQ was developed through a COSMIN-based procedure that reduced an initial pool of 64 candidate questions to the final 23 items, that the developers make a validation manual available on request, and that new language versions are expected to follow the COSMIN protocol and to involve the development team at the translation stage. We state that future Indonesian work should be conducted explicitly against that guidance.

We have been careful not to overstate our own conformity with those recommendations. Our adaptation followed a five-step forward-translation, reconciliation, blinded back-translation, expert-review and cognitive-debriefing procedure as described in the Methods, but as noted above the supporting documentation was not available for this revision and is flagged for author confirmation.

Response to the Journal Requirements

Reference list. We have reviewed the full reference list. We found no retracted or materially corrected references. We made four evidence-based changes, each explained in the claim-and-reference audit accompanying this letter (S4 File):

• Reference 1 no longer supports the opening sentence, because we corrected the global ageing figures. The previous text stated that approximately 720 million people are aged 65 or older and that one in five people worldwide will be older adults by 2030, which mixed the 65+ and 60+ thresholds. The text now uses the WHO figures directly (1 billion people aged 60+ in 2020 rising to 1.4 billion by 2030; one in six people aged 60 or over by 2030), cited to the WHO Ageing and Health fact sheet.

• Reference 27 concerned the Pearson product-moment correlation coefficient but was cited to support a Spearman-based item–total procedure. It has been replaced with de Winter, Gosling and Potter (2016), which directly compares the Pearson and Spearman coefficients.

• Reference 33 concerned the Age-Friendly Environment Assessment Tool, a different instrument, and was cited in support of a statement about cross-cultural validation of the AFCCQ. The sentence has been deleted as peripheral, and reference 33 now cites the Hebrew AFCCQ validation, which is used in the new comparison paragraph.

• References 8, 21 and 41 had incomplete or inaccurate metadata (missing article numbers, an abbreviated author name). These have been corrected and DOIs added, verified against the official AFCCQ page and the publishers' records.

Data availability. The Data Availability Statement has been revised to describe the deposited files accurately, including the fact that geographic information is released only as the five-level urban category, so the city-level counts in S1 Table cannot be regenerated from the public file.

Supporting information. The previous manuscript labelled the Indonesian questionnaire as “S1 File”, but the supplementary file actually supplied is a city/location table. The labels have been corrected: S1 Table is the city distribution table, S2 File is the Indonesian AFCCQ instrument, S3 File is the analysis script and log, and S4 File is the claim and reference audit. [AUTHOR CONFIRMATION REQUIRED] The Indonesian instrument file was not among the materials available for this revision and must be attached before resubmission, or the S2 File reference deleted.

Generative AI disclosure. Our previous statement said that AI was not used to conduct statistical analyses. That statement is no longer accurate for this revision and we have replaced it rather than leave it standing. The revised disclosure states that a large language model was used to write the reproducible analysis script that recomputed the results after the eligibility error was identified, that the authors specified the analysis plan, executed and inspected the code, and independently verified every reported value against the source data and against SPSS output, and that the authors take full responsibility for the integrity and accuracy of all reported results.

Other internal corrections. “Table 54” has been corrected to Table 4 and table and figure numbering checked for sequence. The discrepancy between the 84 municipalities stated in the text and the 93 location labels listed in the supplement has been resolved and explained in S1 Table. Nominal p-values are no longer described as “significant” anywhere in the manuscript, and the Conclusion has been tempered so that it no longer asserts city-category differences that did not survive correction for multiplicity.

We are grateful to the reviewer for comments that led us to re-examine the analytic dataset, and we hope the revised manuscript is now suitable for publication.

Attachment Submitted filename: Response to Reviewers - R2.docx

# Editor report: Decision Letter 2

**DOI:** 10.1371/journal.pone.0358251.r005

**Authors:** Mohammad Saadati

31 Aug 2026

Assessing Age-Friendly Urban Environments in Indonesia: Validation of the Indonesian Age-Friendly Cities and Communities Questionnaire (AFCCQ) and Differences Across Urban City Categories

PONE-D-25-23142R2

Dear Dr. Sari,

We’re pleased to inform you that your manuscript has been judged scientifically suitable for publication and will be formally accepted for publication once it meets all outstanding technical requirements.

Within one week, you’ll receive an e-mail detailing the required amendments. When these have been addressed, you’ll receive a formal acceptance letter and your manuscript will be scheduled for publication.

An invoice will be generated when your article is formally accepted. Please note, if your institution has a publishing partnership with PLOS and your article meets the relevant criteria, all or part of your publication costs will be covered. Please make sure your user information is up-to-date by logging into Editorial Manager at Editorial Manager® and clicking the ‘Update My Information' link at the top of the page. For questions related to billing, please contact billing support.

If your institution or institutions have a press office, please notify them about your upcoming paper to help maximize its impact. If they’ll be preparing press materials, please inform our press team as soon as possible -- no later than 48 hours after receiving the formal acceptance. Your manuscript will remain under strict press embargo until 2 pm Eastern Time on the date of publication. For more information, please contact onepress@plos.org.

Kind regards,

Mohammad Saadati

Academic Editor

PLOS One

Additional Editor Comments (optional):

Reviewers' comments:

# Editor report: Acceptance letter

**DOI:** 10.1371/journal.pone.0358251.r006

**Authors:** Mohammad Saadati

PONE-D-25-23142R2

PLOS One

Dear Dr. Sari,

I'm pleased to inform you that your manuscript has been deemed suitable for publication in PLOS One. Congratulations! Your manuscript is now being handed over to our production team.

At this stage, our production department will prepare your paper for publication. This includes ensuring the following:

* All references, tables, and figures are properly cited

* All relevant supporting information is included in the manuscript submission,

* There are no issues that prevent the paper from being properly typeset

You will receive further instructions from the production team, including instructions on how to review your proof when it is ready. Please keep in mind that we are working through a large volume of accepted articles, so please give us a few days to review your paper and let you know the next and final steps.

Lastly, if your institution or institutions have a press office, please let them know about your upcoming paper now to help maximize its impact. If they'll be preparing press materials, please inform our press team within the next 48 hours. Your manuscript will remain under strict press embargo until 2 pm Eastern Time on the date of publication. For more information, please contact onepress@plos.org.

You will receive an invoice from PLOS for your publication fee after your manuscript has reached the completed accept phase. If you receive an email requesting payment before acceptance or for any other service, this may be a phishing scheme. Learn how to identify phishing emails and protect your accounts at https://explore.plos.org/phishing.

If we can help with anything else, please email us at customercare@plos.org.

Thank you for submitting your work to PLOS One and supporting open access.

Kind regards,

PLOS One Editorial Office Staff

on behalf of

Dr. Mohammad Saadati

Academic Editor

PLOS One
